# Lake Tanganyika—A 'Melting Pot' of Ancient and Young Cichlid Lineages (Teleostei: Cichlidae)?

**DOI:** 10.1371/journal.pone.0125043

**Published:** 2015-04-30

**Authors:** Juliane D. Weiss, Fenton P. D. Cotterill, Ulrich K. Schliewen

**Affiliations:** 1 Department of Ichthyology, Bavarian State Collection of Zoology, Münchhausenstr. 21, 81247 München, Germany; 2 Geoecodynamics Research Hub, c/o Department of Botany and Zoology, University of Stellenbosch, Private Bag X1 Matieland, 7602, Stellenbosch, South Africa; University of Basel, SWITZERLAND

## Abstract

A long history of research focused on the East Africa cichlid radiations (EAR) revealed discrepancies between mtDNA and nuclear phylogenies, suggesting that interspecific hybridisation may have been significant during the radiation of these fishes. The approximately 250 cichlid species of Lake Tanganyika have their roots in a monophyletic African cichlid assemblage, but controversies remain about the precise phylogenetic origin and placement of different lineages and consequently about L. Tanganyika colonization scenarios. 3312 AFLP loci and the mitochondrial ND2 gene were genotyped for 91 species representing almost all major lacustrine and riverine haplotilapiine east African cichlid lineages with a focus on L. Tanganyika endemics. Explicitly testing for the possibility of ancient hybridisation events, a comprehensive phylogenetic network hypothesis is proposed for the origin and diversification of L. Tanganyika cichlids. Inference of discordant phylogenetic signal strongly suggests that the genomes of two endemic L. Tanganyika tribes, Eretmodini and Tropheini, are composed of an ancient mixture of riverine and lacustrine lineages. For the first time a strong monophyly signal of all non-haplochromine mouthbrooding species endemic to L. Tanganyika (“ancient mouthbrooders”) was detected. Further, in the genomes of early diverging L. Tanganyika endemics Trematocarini, Bathybatini, Hemibatini and *Boulengerochromis* genetic components of other lineages belonging to the East African Radiation appear to be present. In combination with recent palaeo-geological results showing that tectonic activity in the L. Tanganyika region resulted in highly dynamic and heterogeneous landscape evolution over the Neogene and Pleistocene, the novel phylogenetic data render a single lacustrine basin as the geographical cradle of the endemic L. Tanganyika cichlid lineages unlikely. Instead a scenario of a pre-rift origin of several independent L. Tanganyika precursor lineages which diversified in ancient rivers and precursor lakes and then amalgamated in the extant L. Tanganyika basin is put forward as an alternative: the 'melting pot Tanganyika' hypothesis.

## Introduction

The ‘Tanganyika Problem’, i.e. the question of the origin of the highly diverse and endemic fauna of Lake Tanganyika (LT), has remained a phylogenetic enigma since Moore (1903) [1]. The arguably most enigmatic faunal element of LT comprises the ~ 250 endemic species of cichlid fishes (Teleostei: Cichlidae). Although it is clear today, that all LT cichlids have their roots in one subgroup of the monophyletic African cichlid assemblage (austrotilapiines) [2], controversies remain about the precise phylogenetic placement and composition of different LT cichlid lineages, which directly reflect on proposed Tanganyika colonization scenarios [3–6]. These controversies result from discordant phylogenetic signal in molecular data sets (e.g. mitochondrial vs. nuclear DNA) and differences in taxon sampling, especially in those phylogenetic analyses that have not included all potentially important cichlid founder lineages of LT. They are further based on substantially different age estimates for critical phylogenetic nodes in the LT cichlid phylogeny, mostly due to molecular clock analyses based on uncertain calibration points.

**Fig 1:**
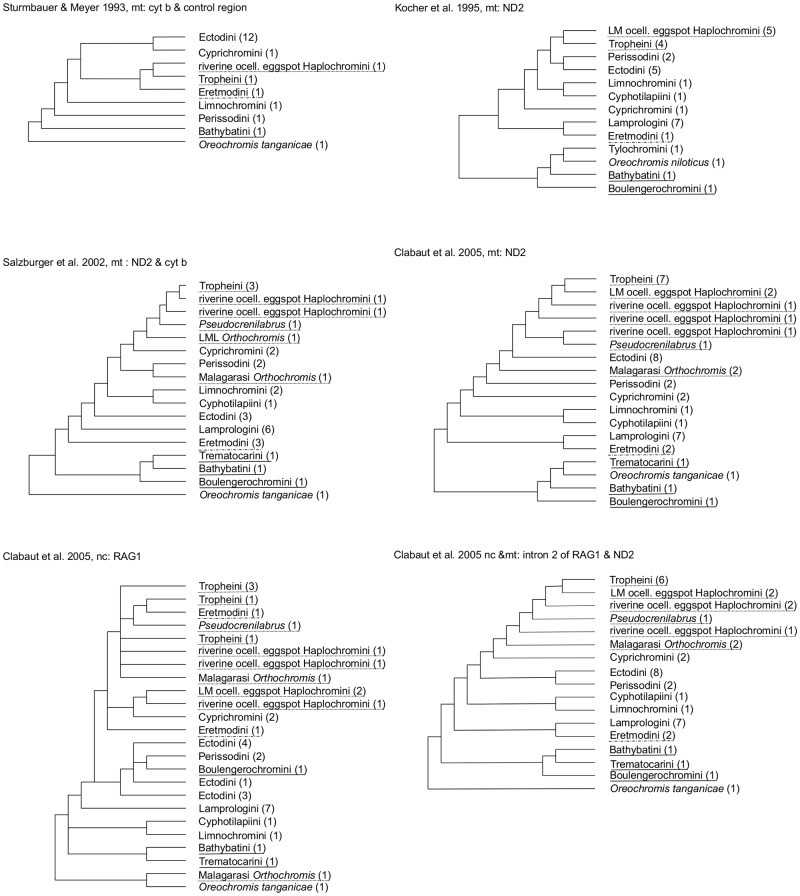
Phylogenetic hypotheses (redrawn) of the relationships among the LT species flock and other representatives of the East African Radiation (EAR). Tribes with ambiguous placement are highlighted as follows: most ancient Tanganyika tribes (continuous line); Eretmodini (dashed line); haplochromine lineages (dotted line). Numbers in brackets correspond to the number of individuals.

The solution of the problem is further impaired by the complexity and age of LT´s ancient cichlid diversity; compared with all other African great lakes, LT hosts both the highest number of endemic cichlid genera and highest number of ancient lineages. Following the pioneering morphology-based classification by Poll 1986 [7] and its revision by Takahashi 2003 [8] the most recent comprehensive classification of LT cichlid genera by Koblmüller et al. 2008 [4] recognizes 13 (almost) endemic tribes, whose monophyly is supported by both molecular and morphological characters: Boulengerochromini, Bathybatini, Hemibatini, Lamprologini, Trematocarini, Eretmodini, Ectodini, Cyphotilapiini, Cyprichromini, Perissodini, Benthochromini, Limnochromini and Tropheini. These are grouped together with additional non-endemic LT cichlid genera into different informally named clades, informed by mitochondrial (mt) and nuclear (nc) evidence (Fig 1) [5,9,10–12]: Takahashi and Okada 2002 [13] suggested Trematocarini, Bathybatini and Hemibatini form the sistergroup to the so called MVhL-clade [14]; this large subgroup—Nishida´s H-lineage [15]—comprises the LT endemic Eretmodini, Limnochromini, Benthochromini, Ectodini, Perissodini, Tropheini, Cyphotilapiini and Lamprologini plus the non-endemic tribe Haplochromini. The latter includes many riverine haplochromine cichlids, as well as >1000 species of the Lake Malawi species flock (LM) and the Lake Victoria superflock (LVSF). The delineation of the H-lineage was based on allozyme data, yet mtDNA data suggested it is better placed with either: (1) Eretmodini as sister taxon, or (2) the remaining MVhL-lineage; or (3) with Eretmodini as sistergroup to Lamprologini (Fig 1) [5,6,9,11,16,17]. Therefore Clabaut et al. [9] in their mtDNA based approach excluded Eretmodini from the MVhL-clade, and named the resulting non-Eretmodini MVhL-clade as C-lineage. Within the latter the rheophilic cichlids of eastern LT affluent drainages Malagarasi, Luiche and Rugufu (here referred to as ‘Malagarasi-*Orthochromis’*) were also identified as an additional distinct clade [6]. The most recent study by Meyer et al. [18] based on an extended nc dataset, recovered Eretmodini as a member of the H-lineage and the sistergroup to the Haplochromini. The composition of all these groups is supported only to a limited extent on both mt and ncDNA data and moreover many potential riverine founder species have not been evaluated; so the phylogenetic integrity of these groupings must still be regarded as preliminary [4].

Absolute and relative age estimates for the origin and radiation of endemic LT cichlid lineages as well as time-calibrated paleo-geomorphological reconstructions of the formation of the extant LT basin are necessary preconditions for the investigation of the ‘Tanganyika Problem’ from the cichlid viewpoint. Current evidence mainly based on mtDNA data [4,5,11] suggests that several precursor cichlid lineages originated from a first major radiation in LT, i.e. the ancestors of *Boulengerochromis*, Hemibatini, Trematocarini, Bathybatini, Eretmodini, Lamprologini and the tribes belonging to the C-lineage as defined by Clabaut et al. [9] based on mtDNA. Subsequent intralacustrine cladogenesis of these ancient lineages is hypothesized to have occurred incrementally in substrate brooding Lamprologini [19,20] and rapidly in non-haplochromine LT mouthbrooding lineages [11,20–24]. The origin of the LT endemic Tropheini is currently debated as they are phylogenetically nested as a comparatively young subclade in the pan-African tribe Haplochromini, which in turn is a composite of riverine and lacustrine lineages. The latter’s phylogeographic history in southern, central and eastern Africa (including the LT region) is complex and only partially understood [25].

Relative age molecular clock estimates of diversification of LT lineages have been based on mtDNA phylogenies only. Nearly all used as calibration points a priori assumptions about the presumed maximum geological age of LT basin formation (12 Ma) and the presumed establishment of true deep- and clearwater conditions of LT (12–6 Ma) [26,27]. These absolute age estimates have to be taken with caution, because fossil or Gondwana fragmentation based calibrations used in large scale cichlid fish phylogenies including a few East African cichlid fishes yield drastically different ages [28–30] (see below, Results and Discussion 3.2.). These analyses revealed that the different LT lineages have different relative node ages of diversification [3,11,19,31]: the early diverging mtDNA lineages leading to *Boulengerochromis*, Bathybatini and Hemibatini, Trematocarini and the subsequently diverging mtDNA precursor lineage of the combined Lamprologini/Eretmodini clade diverged substantially earlier than Lamprologini, Bathybatini and Ectodini. Yet, the most recent common ancestor (MRCA) of the latter mtDNA clade (Ectodini) still appears older than the haplotype diversification age of the remaining mouthbrooding lineages Cyprichromini, Limnochromini, Perissodini, Tropheini and Eretmodini [19]. These mtDNA results presented a simplified reconstruction for the spatio-temporal origin of the LT cichlid flocks, especially when we acknowledge that the geological history of the current LT basin is highly speculative [3,32] (see Discussion below). Since these earlier studies, two new factors further complicate the quest to explain the LT conundrum. The first is the now well documented significant contribution of riverine cichlids to genomes of the lake cichlid species flocks [33,34], and the second is hybridization, which cannot be discarded as a potential driver for the partial origin of species flocks [35]. This is supported by theoretical inferences [36] and the multiple cases of introgressive hybridisation within lacustrine cichlid lineages [18,35,37–45].

Therefore, a molecular phylogenetic hypothesis for the formation and diversification of the entire LT Tanganyika species assemblage must account for: (1) impacts of potential riverine precursor lineages, as well as (2) critically evaluate reticulate phylogenetic signals. Above all, evaluations of all relevant biological factors must critically reexamine the geological history of the LT basin. Here, we revisit this phylogenetic uncertainty of LT cichlids and their extant precursor lineages, which interrelate tightly with ages of LT cichlids and their habitats. Based for the first time on thousands of nc loci and one mtDNA marker, we present a revised reconstruction of LT cichlid relationships comprising important riverine east African cichlids as well as an almost fully representative taxon sampling across all Lake Tanganyika cichlid tribes and genera (Table 1). In order to take into account possible ancient hybridisation events between those cichlid clades [18], that were ambiguously placed in either nc or mtDNA based phylogenetic trees (Fig 1), we explicitly evaluate the validity of non-dichotomous tree hypotheses, i.e. for:

1. phylogenetic relationships of the most ancient LT lineages (tribes), i.e. Boulengerochromini, Trematocarini and Bathybatini (incl. Hemibates) with the remaining EAR and riverine austrotilapiines. Sistergroup relationships of these earliest diverging LT tribes with the remaining EAR lineages have not yet been established with confidence and are partially contradictory in different studies [5,9,16,18,29,46] (Fig 1), and no study has yet included all of the aforementioned LT tribes as well as all riverine austrotilapiine lineages, despite the fact that the latter represent the sistergroup to the EAR [2,29].
2. phylogenetic relationships of LT Eretmodini with haplochromines including *Orthochromis* and non-haplochromine LT mouthbrooding lineages (Cyphotilapiini, Limnochromini, Perissodini, Cyprichromini, Ectodini and Benthochromini). Clabaut et al. [9] as well as Meyer et al. [18] revealed major discrepancies between their mt and nc phylogeny (Fig 1) raising the question of a mosaic genomic structure of Eretmodini.
3. phylogenetic relationships of LT Tropheini with other Haplochromini lineages from eastern, central and southern Africa, i.e. lacustrine and riverine Haplochromini with ocellated eggspots (“modern” Haplochomini), *Pseudocrenilabrus*-related cichlids, serranochromines and the rheophilic *Orthochromis* from northern Zambia, Luapula-Mweru-Lualaba and Malagarasi drainage basins. Depending on the applied marker technique different sistergroup relationships among these lineages were recovered in [6,9,16,17] (Fig 1), and results of [25,33,34] and [47] have suggested that different riverine haplochromine lineages could have served as 'transporters for genomic variation' when they hybridized with other lineages, possibly affecting Tropheini.

**Table 1:** Overview of nomenclature, group affiliation and sampling.

East African Radiation (EAR) tribes	Distribution	Group names used in this study	MVhL lineage [14]	H-lineage [15]	C-lineage [9]	Genera/Species included in this study
Boulengerochromini	Lake Tanganyika	'most ancient Tanganyika tribes'				*Boulengerochromis (1)*
Bathybatini incl. Hemibatini	Lake Tanganyika	'most ancient Tanganyika tribes'				*Bathybates*, *Hemibates (2)*
Trematocarini	Lake Tanganyika	'most ancient Tanganyika tribes'				*Trematocara (2)*
Lamprologini	Congo basin incl. Lake Tanganyika & Lower Malagarasi River	Lamprologini	x			*Lamprologus*, *Altolamprologus*, *Neolamprologus*, *Lepidiolamprologus*, *Variabilichromis*, *Chalinochromis*, *Julidochromis (12)*
Eretmodini	Lake Tanganyika	Eretmodini	x	x		*Eretmodus*, *Spathodus*, *Tanganicodus (4)*
Ectodini	Lake Tanganyika	'ancient Tanganyika mouthbrooders'	x	x	x	*Xenotilapia*, *Grammatotria*, *Callochromis*, *Opthalmotilapia*, *Aulonocranus*, *Cyatopharynx (7)*
Cyprichromini	Lake Tanganyika	'ancient Tanganyika mouthbrooders'	x	x	x	*Cyprichromis*, *Paracyprichromis (2)*
Perissodini	Lake Tanganyika	'ancient Tanganyika mouthbrooders'	x	x	x	*Perissodus*, *Haplotaxodon (2)*
Limnochromini	Lake Tanganyika	'ancient Tanganyika mouthbrooders'	x	x	x	*Limnochromis*, *Gnathochromis*, *Triglachromis*, *Reganochromis (4)*
Cyphotilapiini	Lake Tanganyika	'ancient Tanganyika mouthbrooders'	x	x	x	*Cyphotilapia (2)*
Benthochromini	Lake Tanganyika	'ancient Tanganyika mouthbrooders'	x	x	x	*Benthochromis (2)*
*'Ctenochromis'*	Lake Tanganyika	'ancient Tanganyika mouthbrooders'	x	x	x	*'Ctenochromis' benthicola (1)*
Tropheini	Lake Tanganyika	Tropheini (= Haplochromini-subgroup)	x	x	x	*Tropheus*, *Petrochromis*, *Interochromis*, *Simochromis*, *'Ctenochromis' horei (6)*
Lake Malawi Haplochromini	Lake Malawi	'ocellated eggspot Haplochromini' (= Haplochromini subgroup)	x	x	x	*Melanochromis*, *Rhamphochromis*, *Pseudotropheus*, *Sciaenochromis*, *Aulonocara*, *'Haplochromis' callipterus (7)*
riverine Haplochromini	Eastern & Central Africa	'ocellated eggspot Haplochromini' (= Haplochromini subgroup)	x	x	x	*Haplochromis*, *Astatoreochromis*, *'Haplochromis' flaviijosephi*, *'H*.*' desfonaini*, *'H*.*' paludinosus (6)*
serranochromines	Southern & Central Africa incl. Southeastern Congo basin	Serranochromines (= Haplochromini subgroup)	x	x	x	*Serranochromis*, *Pharyngochromis*, *'Orthochromis' torrenticola*, *'Haplochromis' fasciatus*, *'H*.*'* sp. 'Kwango', *Cyclopharynx*, *Haplochromis*, *'Pharyngochromis'* sp. 'white tip', *'Pharyngochromis'* sp. 'yellow lips' *(12)*
*Orthochromis*	northern Zambia	Northern-Zambian-*Orthochromis*	x	x	x	*'Orthochromis' kalungwishiensis*, *'O*.*'sp*.*aff*. *kalungwishiensis (2)*
	Luapula-Mweru system & Lualaba/Congo mainstem	'LML-*Orthochromis'*	x	x	x	*'Orthochromis' polyacanthus*, *'O*.*' stormsi (2)*
	Malagarasi River	'Malagarasi-*Orthochromis'*	x	x	x	*Orthochromis (2)*
*Pseudocrenilabrus*	Southern Africa, Southern & Eastern Congo basin, Nile drainage	'Pseudocrenilabrus-group' (= Haplochromini subgroup)	x	x	x	*Pseudocrenilabrus (4)*
	northern Zambia	'Pseudocrenilabrus-group' (= Haplochromini subgroup)	x	x	x	'New Kalungwishi cichlid' (1)

## Material and Methods

### 2.1. Sampling

94 individuals representing 91 haplotilapiine cichlid species (*sensu* Schliewen and Stiassny) [43] were included to represent all major lacustrine and riverine cichlid clades represented in the East African Radiation (EAR; Table 1; voucher information see S1 Table). Based on recent phylogenetic results of [2,49] the mouthbrooding oreochromine cichlid *Oreochromis tanganicae* (N = 2) and the substrate brooding coptodonine *Coptodon rendalli* (N = 1) were used in all analyses as outgroups for the ‘austrotilapiine’ ingroup (sensu Schwarzer et al.) [2], which consists of riverine, substrate brooding cichlid clades on the one hand and the EAR on the other hand. Concerning the first, all substrate brooding riverine genera with N = 9 species and N = 9 samples of the genera *Steatocranus* s.str., *Tilapia* s. str. *Chilochromis* and *Congolapia* were included in this study. The EAR was represented by N = 81 species with N = 83 samples comprising the following clades and genera, completely incorporating all described Tanganyika cichlid tribes recently revised by Takahashi [8] and modified by Koblmüller et al. [4], whose tribal nomenclature we follow here: The four anciently diverging Tanganyika genera *Boulengerochromis* (Boulengerochromini), *Bathybates*, *Hemibates* and *Trematocara*; L. Tanganyika and Congo basin substrate brooders of genera *Lamprologus*, *Neolamprologus*, *Lepidiolamprologus*, *Altolamprologus*, *Chalinochromis*, *Variabilichromis*, *Telmatochromis* and *Julidochromis* (N = 11 (Lamprologini). Ancient Tanganyika mouthbrooders (N = 20) of the genera *Callochromis Cyathopharynx*, *Aulonocranus*, *Opthalmotilapia*, *Grammatotria*, *Xenotilapia* (Ectodini); *Limnochromis*, *Gnathochromis*, *Triglachromis*, *Reganochromis* (Limnochromini); *Cyprichromis* and *Paracyprichromis* (Cyprichromini), *Benthochromis* (Bethochromini), *Perissodus*, *Haplotaxodon* (Perissodini); *Cyphotilapia* and ‘*Ctenochromis*‘ *benthicola* (according to Muschick et al. [46] and our own data member of Cyphotilapiini). Lake Tanganyika Eretmodini (N = 4) of the genera *Spathodus*, *Tanganicodus* and *Eretmodus*. Riverine taxa were represented by ‘Malagarasi-*Orthochromis*’ (N = 2), i.e. *Orthochromis malagaraziensis* and *Orthochromis uvinzae; Pseudocrenilabrus* (N = 4) and one member of a yet undescribed genus (‘New Kalungwhishi cichlid’). ‘*Orthochromis*‘ from the Luapula-Mweru-Lualaba drainage (here treated from now on as ‘LML-*Orthochromis’*; N = 2), i.e. ‘*O*.‘ *stormsi*, ‘*O*.‘ *polyacanthus; Orthochromis* from northern Zambia (here treated from now on as ‘northern-Zambian-*Orthochromis’*; N = 2), i.e. ‘*O*.‘ *kalungwishiensis*, ‘*O*.‘ sp. aff. *kalungwishiensis*. Serranochromines and related taxa from southern and central Africa of the genera *Serranochromis*, *Pharyngochromis*, *Cyclopharynx* and ‘*Haplochromis*‘ (Congo) and ‘*Orthochromis*‘ *torrenticola* (N = 12). Lake Tanganyika Tropheini were represented by the genera *Tropheus*, *Interochromis*, *Petrochromis*, *Simochromis*, *and* ‘*Ctenochromis*‘ *horei* (N = 6), and the East African Haplochromini (N = 13) by the genera *Astatoreochromis*, ‘*Haplochromis*‘, *Rhamphochromis*, *Melanochromis*, *Pseudotropheus*, *Sciaenochromis* and *Aulonocara*. Acknowledging taxonomic uncertainty in placing haplochromine species in the genera of Greenwood [50,51], we follow the pragmatic approach first suggested by Hoogerhoud [52], i.e. to place taxa of doubtful generic status in the catch-all genus ‘*Haplochromis*‘, or analogously in ‘*Orthochromis* or ‘*Ctenochromis’* (in quotation marks).

Numbers in brackets correspond to the number of individuals included in this study.

The manuscript was prepared strictly adhering to the PLOS Data Policy, i.e. genbank accession numbers are provided for ND2 sequences (256013278, 256013292, 256013302, 256013304, 407729195, 407729205, 407729221, 407729223, 407729291, 407729297, 407729299, 478445907, 478445913, 478445927, 478445989, KJ176277, KJ176278, KJ176279, AF317267.1, AY337769.1, AY337794.1, AY602994.1, AY682542.1, AY682544.1, AY740339.1, AY930048.1, AY930056.1, AY930069.1, AY930094.1, EF393712.1, EF679251.1, EF679255.1, GQ167833.1, GQ995761.1, GQ995809.1, HM623786.1, KJ176256, KJ176259, KJ176262, KJ176264—KJ176267, KJ176270—KJ176272, KJ176280—KJ176282) and access to the AFLP datamatrix is gained via doi:10.5061/dryad.8k0g5.

#### 2.1.2. Ethics statement

Most fish specimens for this study were obtained from the commercial cichlid fish trade in Germany, and all specimens were obtained prior to legal implementation of the Nagoya Protocol (NP) on October 12^th^ 2014. For aquarium stocks that originate and have been bred in Germany, German and European Access and Benefit Sharing (ABS) law applies. The German ABS law is not submitted yet, and the European law does not restrict access to genetic resources originating from the European Community. Nevertheless we checked whether national legislation for access and utilization of samples apply for countries where traded specimens or their offspring may have ultimately derived from (n/a = not applicable): Tanzania (n/a), Sambia (n/a), DRC (n/a), Namibia (Oct 12 2014), Angola (n/a), Egypt (Oct 12 2014), South Africa (Oct 12 2014), Malawi (Nov 24 2014), Burundi (Oct 12 2014); see http://www.cbd.int/abs/nagoya-protocol/signatories/default.shtml. Research permits for the wild collection and export of Congolese fishes were granted under DRC Research Permit No. AC/113/2013/I.S.P./MBNG/AUT.AC (issued by the Republique Democratique du Congo, Institut Superieur Pedagogique de Mbanza-Ngungu, confirmed by the Ministries of the Interior and Agriculture Direction Provinciale du Bas-Congo, Dem. Rep. du Congo). Sampling in the wild did not include or effect any internationally protected species. None of the species is listed as protected under CITES, European or German law (compare http://www.wisia.de/FsetWisia1.de.html -> Global query for all fishes and lampreys). See above and compare http://www.wisia.de/FsetWisia1.de.html (-> Global query for all fishes and lampreys). In addition, sampling in DRC was supervised by Paul N’lemvo Budiongo, Institut Congolais pour la Conservation de la Nature (ICCN).

Work protocols for sacrificing fish specimens comply with the German Tierschutzgesetz (TSchG), especially §2 (rearing), § 7a(1)6. Further, all sampling procedures have been reviewed prior to sampling and sampling was thoroughly planned complying with Neumann 2010 [53]. Specimens were processed according to procedures in Neumann 2010 [53], i.e. specimens were narcotised in an overdosed approved fish anaesthetic (Benzocaine, MS-222) until they were dead and only thereafter then tissue sampled. Specimens collected in the wild (DRC) were caught using rotenone or bought already dead from local fishermen. As soon as specimens appeared at the water surface, they were collected into buckets with overdosed Benzocaine and processed as explained above [53]. No separate ethical approval for the animal use for this research was necessary, because (1) scientists and technical staff of ZSM have not performed experiments concerning EU directive 2010/63/EU, especially those laid down in the General Provisions of Article 1 §1 (use of animals for scientific and educational purpose) and §2 (animals used or intended for use in procedures), (2) our studies on cichlid diversity in the Congo basin are explicitly exempted and thus do not fall under regulations of EU directive 2010/63/EU (see Article 1 §5.e)), and (3) the work and scientific research carried out by scientist and technical staff of our institutions fully comply with the German Tierschutzgesetz (TSchG), and neither from the EU directive 2010/63/EU nor from the TSchG an Animal Care and Use Committee a separate statement is necessary according to current German law (as it might be stipulated from US law).

### 2.2. Molecular Methods

Total genomic DNA was isolated from muscle tissue or fin clips using the DNeasy Blood and Tissue Extraction Kit (Quiagen) following the manufacturer´s protocol. AFLP data were generated using a modification of the method [54] as applied by Herder et al. [55]. The following 20 *MseI/EcoRI* primer pairs were used for selective amplification of fragments: AGG*-CTG; ACA*-CAA; ACA*-CTG; ACT*-CAA; AGG*-CTC; ACC*-CTA; ACT*-CAG; ACC*-CAT; AGG*-CTA; ACA*-CAT; ACT*-CTG; ACC*-CAG; ACT*-CTT; AGC*-CTC; AGG*-CAA; AGC*-CAC; AGG*-CTT; AGC*-CAG, ACT*-CAC, ACC*-CTC. Bands were visualized on an AB 3130 sequencer (Applied Biosystems) with size standard ROX 500XL. The Genemapper v. 4.0. software (Applied Biosystems) was used for automatically scoring peaks between 50 and 499 bases for presence/absence. Three individuals were double genotyped to assess error rates. To correct for standard error of automated sequencers a bin correction was applied to the dataset following Schwarzer et al. [29].

Partial mitochondrial ND2 sequences were generated using primers ND2Met and ND2Trp for amplification [16]. These new ND2 sequence data were combined with published sequences of all other species taxa represented in the AFLP data set except for ‘*Haplochromis*‘ *flaviijosephii*, where amplification repeatedly failed, yielding a data set of 90 terminals.

### 2.3. Phylogenetic Inference and Detection of Hybrid Signal

#### 2.3.1. General approach

A complementary approach was adopted to establish a first reticulate phylogenetic hypothesis for LT cichlids:

1. Independent phylogenetic analysis of each a single locus mtDNA and a multilocus AFLP data set, i.e. calculation of dichotomous tree hypotheses. To assess the phylogenetic consistency of Neighbour Joining (NJ)-based AFLP based hypotheses, which are the basis for Homoplasy Excess Tests (HET; see below), NJ cladograms were compared with Bayesian Inference (BI) and Maximum Parsimony (MP) based cladograms.
2. Identification of cyto-nuclear discordance of strongly supported nodes, potentially indicating ancient or ongoing interspecific gene flow with focus on the three ambiguous phylogenetic relationships which were conspicuous in several previous studies (Fig 1, see Introduction).
3. Homoplasy Excess Tests (HETs) [36] were executed for the differential inference of potential hybrid signal with respect to contribution of selected parental taxa from within the EAR and potential founder lineages. HET is a tree based method which has been successfully used for identifying potential hybrid taxa through their ‘homoplasy’ effects on statistical support for a particular node in a dichotomous phylogenetic hypothesis [25,38,49]. Based on the prediction that hybrid taxa weaken statistical support values for particular nodes due to the mosaic nature of their genome, parental taxa are inferred through the effects of taxon removals, where only the removal of putative hybrid taxa should lead to significant increase of BS support values for nodes placing parental taxa with their sister taxa [36]. Theoretically, homoplasious effects could also result from ancient shared polymorphisms due to incomplete lineage sorting. With HET, the distinction between hybridisation and incomplete lineage sorting is possible also in large multi-taxon phylogenies because incomplete lineage sorting should rather lead to randomly dispersed homoplasy (taxon removal) effects across the ingroup nodes of a dichotomous tree hypothesis, whereas a non-random distribution of increased BS support effects at particular nodes is unlikely for incomplete lineage sorting [36].
4. Non-metric multidimensional scaling (nMDS) of presence/absence AFLP data was used as a phylogenetically independent means to assess the validity of mtDNA and ncDNA based phylogenetic hypotheses including results from HET indicating ancient gene-flow. nMDS of presence/absence AFLP data allows for a NJ-independent investigation of genomic similarity and intermediacy of species and species groups in multilocus data sets as reflected by distances in bivariate plots. This method is especially suitable for AFLP data because it allows using Jaccard´s distances (as in HET) [62] when iterative positioning in space is performed towards the global minimum of individual stress-values, and because nMDS does not assume linear or modal correlations in the data set. By performing multiple reduced nMDS projections, in which major sistergroups of ingroup taxa as identified in the consensus NJ AFLP topology are stepwise eliminated, genomic similarities of species or species groups as well as intermediacy of hypothetical hybrid taxa or clades of hybrid origin (as identified as candidates by cyto-nuclear discordance and HET) can be inferred in a stepwise altered variance space.
5. Finally, a split-based NeighborNet depicting conflicting signal in the AFLP dataset was constructed to assess consilience with inferred gene-flow patterns derived from HET and nMDS. The network construction is again based on Jaccard´s distances [62], i.e. neglecting absence of bands, which cannot be unambiguously interpreted as loss of a particular restriction site, and therefore the implemented algorithm can be referred to as conservative. A hybrid taxon is expected to be at the intersection of two parental splits [57].

#### 2.3.2. Technical details of analytical methodology

1. MEGA v. 5 [58] was used to build a multiple alignment of partial ND2 sequences (1010bp) applying the ClustalW algorithm and to calculate nucleotide frequencies. Codon positions were checked separately for saturation by calculating the absolute number of transitions/transversions using PAUP v. 4.0 [59] and plotting them against each other. A maximum likelihood analysis (ML) was perfomed with RAxML v. 7.2.6 [60] using the GTR+Г model and the rapid bootstrap algorithm [61] with a subsequent search for the best-scoring ML tree. In addition, 500 bootstrap (BS) pseudo-replicates were calculated for evaluation of branch support. For the nc AFLP dataset a NJ tree based on Jaccard´s distances [62] was calculated with 500 BS replicates using TREECON v. 1.3b [63]. Since distance based clustering methods like NJ may suffer from the drawback that the topology is calculated based on a distance matrix rather than on character state transition probabilities, i.e. their results rather reflect overall similarity and not necessarily phylogenetic relationships, the NJ analysis of the full AFLP data set was complemented with Maximum Parsimony (MP) and a MCMC Bayesian phylogenetic inference (BI) in order to evaluate the phylogenetic validity of the NJ topology. MP analysis was performed in PAUP version 4.0b10 (Altivec) [59] as a heuristic search comprising 1000 random additional sequences keeping one tree at each step. Branch support values were determined with a bootstrap analysis comprising 1000 replicates and a final 50%majority rule consensus topology was calculated from these replicates. BI was conducted with MrBayes v3.2.2 [64] using the restriction site binary model nst = 1 with “no absencesites”, i.e. a simple F81-like model [65] only taking into account the asymmetry in band gains and losses [66]. BI was conducted with 6000000 generations, a relative burnin of 25% and with report of convergence diagnostics to assure a good sample from the posterior distribution based on the Potential Scale Reduction Factor (PSRF). Effective sample size (ESS) calculated with Tracer v.1.6.0 [67] was also used to assure sufficient sampling and convergence of chains.
2. Alternative dichotomous phylogenetic hypotheses of maternally inherited mtDNA (ND2 dataset) and the multilocus (AFLP) nc dataset were evaluated for significant differences between cyto-nuclear discordant topologies with Approximately Unbiased (AU) tests [68] using CONSEL v. 0.1 [69].
3. Our experimental HET design of the AFLP dataset contained consecutive full and partial taxon removal experiments of every single individual (N = 93; except for the outgroup taxa), as well as of combinations of taxa representing all major clades (N = 64). Each removal experiment was duplicated to evaluate algorithm variance, i.e. the total number of removal experiments was 314. As for the complete dataset, for each of these 314 datasets a NJ tree based on Jaccard´s distances [62] was calculated with 500 BS replicates using TREECON v. 1.3b [63], and BS support for all identical nodes was reported manually. If removals resulted in alternative majority rule BS topologies of clades/taxa in the consensus tree, the BS support value for the original node present in the all taxon consensus topology was calculated using the option 'tree support' implemented in Phyutility v. 2.2 [70]. Importantly, to evaluate whether a given increase in BS support was due to a non-random effect we performed additional 100 taxon-removal experiments of each 11 randomly chosen taxa in our dataset, because N = 11 corresponded to the largest number of taxa that were included in the collective removal experiments of taxa of major clades. For these 100 removals calculation of distance trees and inference of BS support values was conducted analogous to the first 314 reduced datasets. We then merged BS support values of both datasets and constructed for each node a schematic boxplot sensu Tukey [71] to assess whether strongly increased BS support values occurred after the removal of particular taxa or clades. Applying a highly conservative approach, only removal effects with the following excess-characteristics identified through the boxplots were deemed non-random effects and are discussed in the following; for doing so only nodes with BS “extreme outlier” values sensu Tukey [71] were considered, i.e. values of both replicate removals were outside the 3-times interquartile range (IQR) of all other values for a given node after all targeted 314 taxon removal and all 100 random taxon removal experiments. Based on the BS values of all removal experiments, a heatmap was generated highlighting all extreme outlier effects at affected nodes. In addition, sistergroup relationships between major clades with BS<80 support in the consensus tree were checked for alternative topologies, because partitioning of alternative topologies may be bimodal and hence may indicate a second dominant alternative topology as opposed to random topologies. Further, when interpreting homoplasious effects special attention was paid to a possible artifact which we term ‘support carryover’, which denotes an artificial increase of BS support at a certain node after removal of an adjacent node with high BS support. This effect is theoretically plausible but surprisingly it is practically not evident in any expected case. Nevertheless potentially affected BS increases should be interpreted with caution and are therefore separately highlighted in our heatmap. Unfortunately HET suffers from saturation of BS values at 100 because the prevention of further BS increase above BS 100 might conceal potential hybrid signal. We point out, that random bias on BS support values of interior nodes introduced by distant outgroups in AFLP data sets as inferred for LT cichlids in Kirchberger et al. [72] are unlikely to produce extreme outliers in our HETs, because of a nested outgroup design, and because of a threefold higher data density in our study. Further, we are extremely conservative with interpretation of HET results and therefore contrasted HET results with two methodologically independent evaluation methods, i.e. non-metric multidimensional scaling (nMDS), and a NeighborNet of AFLP data.
4. nMDS of presence/absence AFLP fragments was conducted as implemented in PAST v. 2.13 [73]. The minimal number of dimensions for the appropriate reflection of data was chosen based on Kruskal´s stress-value, which reflects how well data ordination summarizes observed distances among the samples, and the 1% cutoff value [74], indicating the 1% confidence threshold of Kruskal´s stress-value for a non-random projection of the given number of samples. By performing multiple reduced nMDS projections, in which major sistergroups of ingroup taxa according to the consensus NJ topology were stepwise eliminated, first the early diverging sistergroups and then increasingly closely related sistergroups, genomic similarities of species or species groups could were inferred in a stepwise altered variance space.
5. A split-based NeighborNet was constructed based on Jaccard´s distances [62] using SplitsTree4 v. 4.12.3 [56].

## Results and Discussion

AFLP genotyping resulted in 3312 loci containing 3282 polymorphic and 2861 parsimony informative sites. BI showed optimal convergence for all model parameters (PSRF+ = 1) after the burnin, while ESS calculated in Tracer indicated sufficient sampling and acceptable mixing (ESS>16000). The NeighborNet illustrates strong recovery of phylogenetic signal (Lsfit = 99.95). The mitochondrial ND2 locus alignment (1010bp) had 543 variable sites with relative nucleotide frequencies T = 0.27, C = 0.35, A = 0.26, G = 0.12.

### 3.1. Dichotomous and reticulate phylogenetic signal of LT cichlids

#### 3.1.1. Strongly supported terminal clades are congruent with previous hypotheses

**Fig 2:**
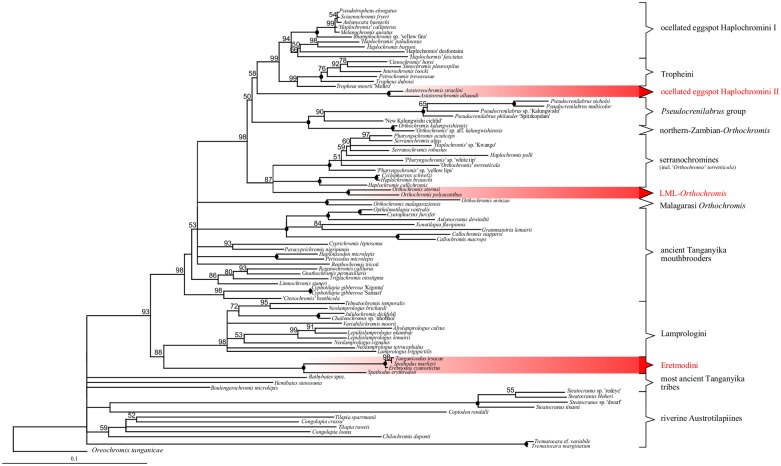
ML- phylogeny of mt sequence data and significant cyto-nuclear discordances. The topology of the best scoring ML tree is based on ND2 sequences (1010bp). Numbers at nodes refer to bootstrap-values (BS 500 replicates), 100% BS support is indicated by filled, black circles. Significant deviations from the nc-based NJ-topology are highlighted in red.

**Fig 3:**
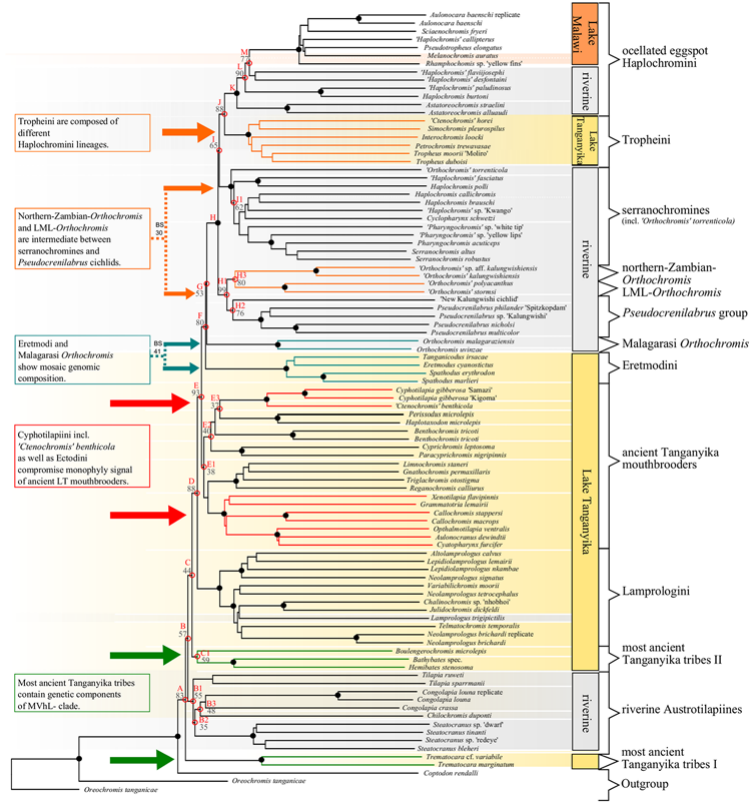
NJ-consensus phylogeny of AFLP data and major effects revealed with HET. The NJ consensus topology is based on Jaccard’s distances [62] of 3312 nc loci. Nodes affected by homoplasious effects are designated with letters A-M and indicated by open, red circles. Numbers at nodes refer to bootstrap-values (BS 500 replicates) and a 100% BS support is indicated by filled, black circles. Geographic distribution of taxa is depicted vertically on the right and colour shaded in the tree (Lake Tanganyika: yellow; Lake Malawi orange; rivers: grey). Major effects detected with HET and inference of cyto-nuclear discordances are delineated in coloured boxes on the left and correspond to those in Fig 6 and Fig 8. Arrows and coloured branches point to clades which especially introduce homoplasy in the dataset. Strong alternative signal (BS support >30) is denoted with dotted lines on the left.

**Fig 4:**
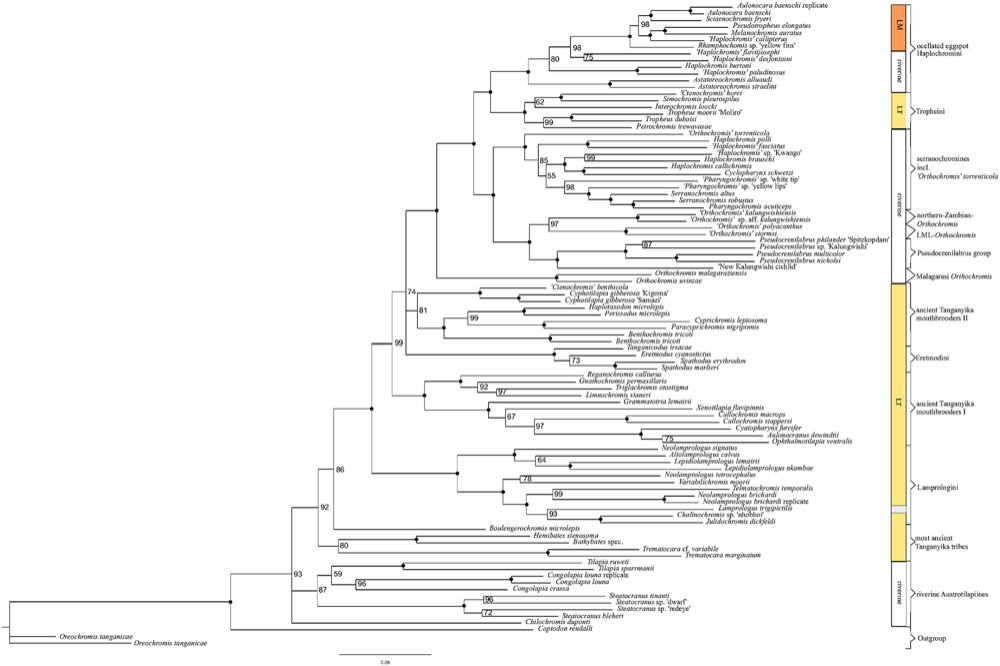
MCMC Bayesian Inference (BI) phylogeny of AFLP data. Consensus topology with branch support values depicted at nodes, dots correspond to 100% Bayesian Posterior Probability.

**Fig 5:**
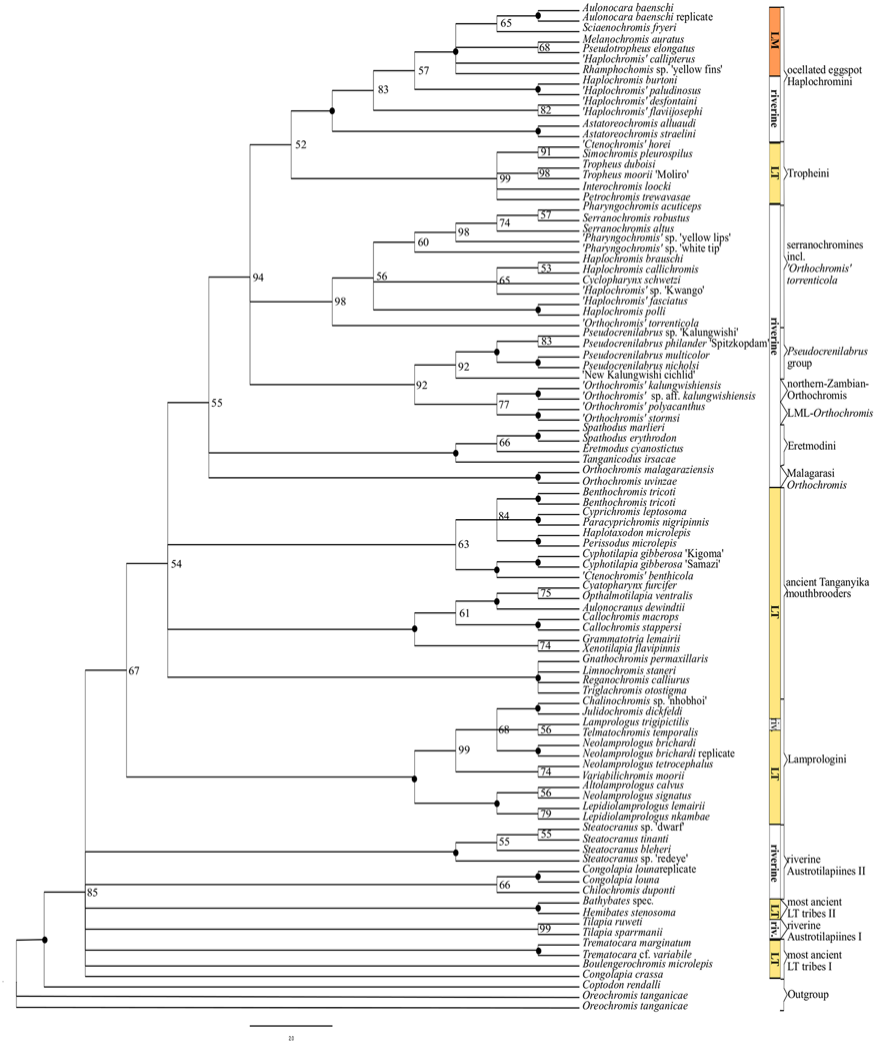
Maximum parsimony (MP) phylogeny of AFLP data. 50% majority rule consensus topology of 1000 BS replicates. BS support is depicted at respective nodes, dots correspond to 100 BS support.

The mtDNA (ND2) hypotheses (Fig 2) and the nc marker (AFLP) phylogenetic hypothesis presented here (Figs 3, 4 and 5) were strongly supported in all analyses (i.e. BS 100 in NJ, BI and MP) concurring with the previously supported (mtDNA and ncDNA) monophyly of major terminal clades Steatocranini [49], Bathybathini together with Hemibatini [11], Trematocarini [46,49,75], Lamprologini [5,6,9,43,45,46,49,76], Ectodini [5,9,17,23,46,49], Cyprichromini [5,21,46,49], Limnochromini [5,6,22,46,70,77], Benthochromini [46,49,78], Perissodini, Eretmodini [5,49] and Cyphotilapiini including “*Ctenochromis” benthicola* [62], and the ‘Malagarasi-*Orthochromis’* [6,49]. Haplochromini are strongly supported too, and are further subdivided into previously recognized subclades Tropheini (NJ, BI & MP: BS 100,ML: BS 99) incl. “*Gnathochromis” pfefferi* [5,38,49,79], a lineage of *Pseudocrenilabrus* including an undescribed species and genus ‘New Kalungwishi cichlid’ (NJ: BS 76, MP: BS 92, BI: BS 100, ML: BS 90) as well as ‘LML-*Orthochromis*’ and ‘northern-Zambian-*Orthochromis’* (both BS 100 in NJ, BI, MP & ML); a group comprising Congo-basin and south-central African (serranochromines) cichlids including *‘Orthochromis’ torrenticola* (NJ & BI: BS 100, MP: BS 98 ML: BS <50 phylogeny; CSA-lineage of [6,25,80,81,] the modern haplochromines with ocellated eggspots (NJ, BI & MP: BS 100, ML: BS 99) [6], and *Astatoreochromis* (NJ, BI, MP & ML BS 100) [6]. Only the monophyly of Tilapiini (sensu Dunz and Schliewen) [49] is poorly supported (ncDNA) and unresolved for Tilapiini genera (mtDNA).

**Fig 6:**
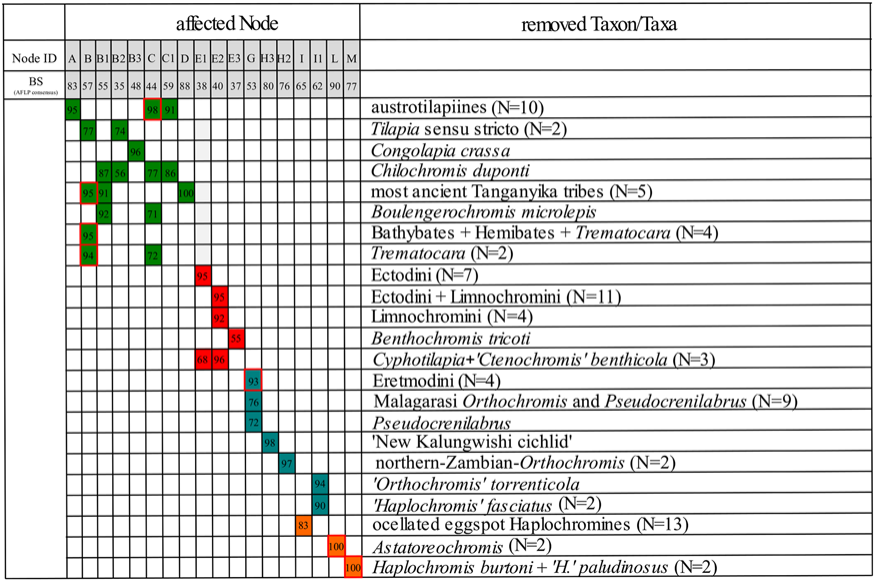
Overview of all removal experiments with major effects as detected with HET. For all affected nodes the bootstrap (BS) support in the consensus AFLP topology as well as BS support after the influential removals is shown and removed taxa are specified. Node IDs correspond to those in the nc NJ-consensus phylogeny (Fig 3). Single effects were grouped according to four major effects and are represented by different colours. Potentially artificial BS increases due to ‘support carryover’ (see methods) are highlighted with red frames.

**Fig 7:**
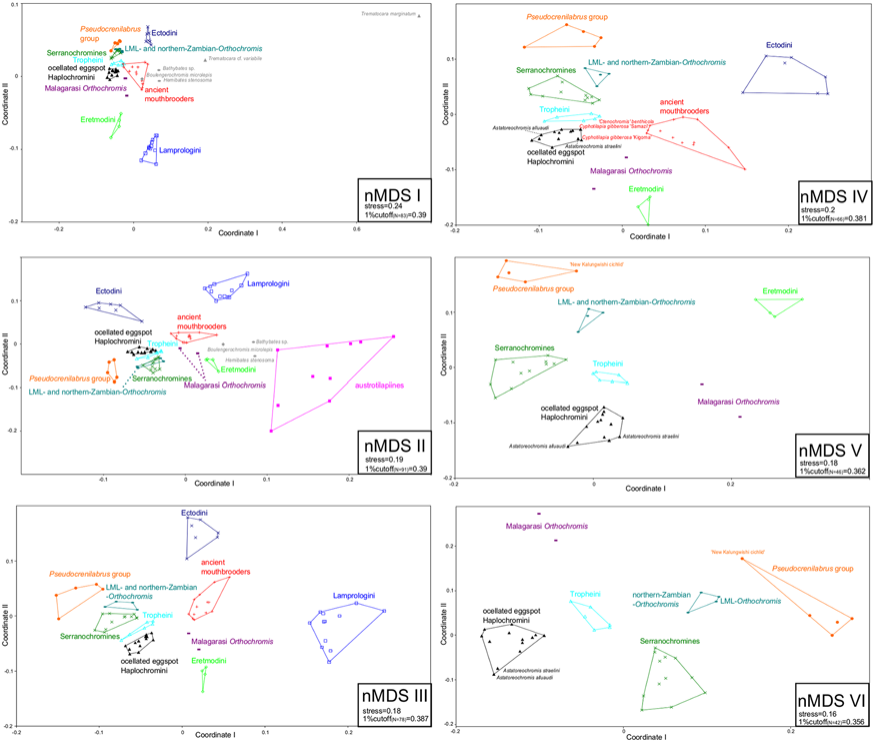
Stepwise reduced nMDS plots. To infer phylogenetic relationships in an altered variance space, major sistergroups of ingroup taxa according to the consensus NJ topology were stepwise eliminated. NMDS plots are based on Jaccard´s distances [62] of nc (AFLP) data. Kruskall’s stress values as well as the corresponding 1% cutoff values [74] are given for each projection.

**Fig 8:**
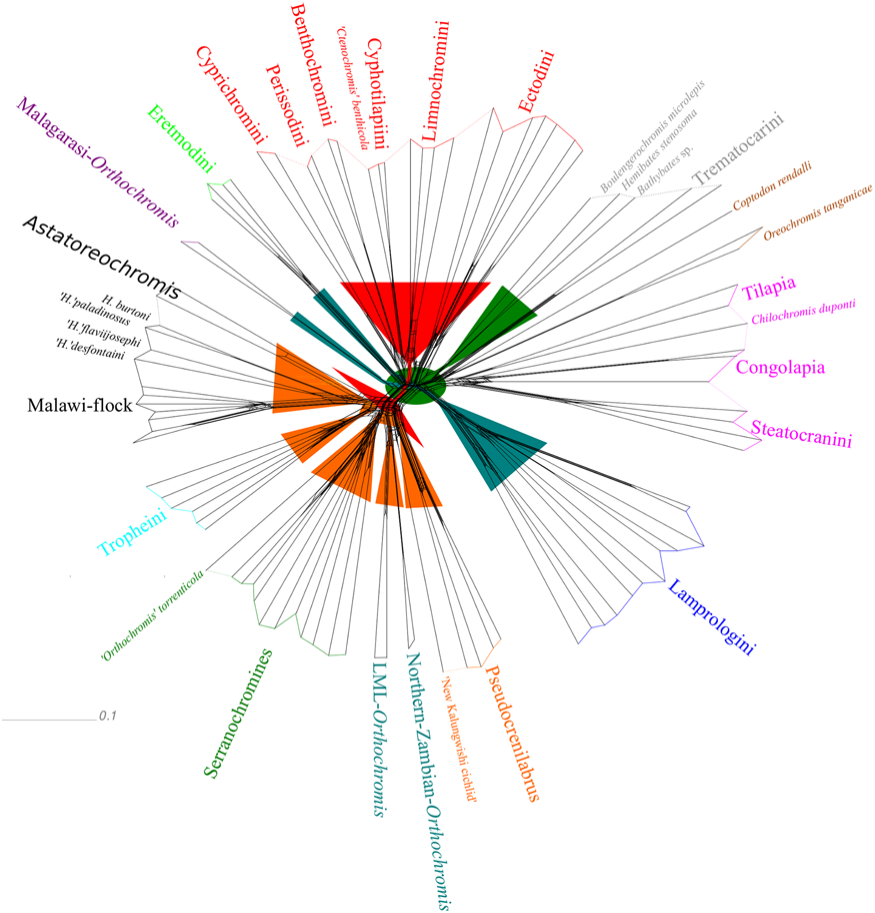
NeighborNet projection based on AFLP data and major effects of HET. The NeighborNet network topology is based on Jaccard’s distances [62]. Specimens of distinct lineages are grouped and informal groups used in this work are depicted in different colours. Funnels highlight conflicting signal corresponding to the four major effects detected with HET and inference of cyto-nuclear discordances and are coloured according to Fig 3 and Fig 6.

Consensus phylogenetic hypotheses derived from nc data (AFLP) with different algorithms (Figs 3,4 and 5) showed a high level of congruence with regard to the interrelationships of the major terminal clades mentioned in 3.1.1, except for differences in BS support values and resolution. Nevertheless, most basal nodes remained comparatively poorly supported in the majority rule phylogenetic hypotheses, but our increased taxon and nucleotide sampling as well as the application of the stepwise analysis of the AFLP data allowed for inference of phylogenetic signal hidden behind poorly supported nodes and was able to support results from previous studies (Fig 1) with regard to cyto-nuclear discordance as well as to establish novel phylogenetic hypotheses. Especially the analysis of results derived from HET (Fig 6) enabled us to infer the presence of reticulate phylogenetic signal in the AFLP dataset, which was further supported by the consecutive comparisons of the obtained HET results with nMDS (Fig 7) and the NeighborNet (Fig 8). The five major results indicating reticulate phylogenetic signal, not evident in the majority rule phylogenetic hypothesis (Fig 3), are highlighted in boxes to the left of respective nodes in Fig 3, and these are discussed in detail below.

#### 3.1.2. Relationships between most ancient Tanganyika tribes, riverine austrotilapiines and the remaining EAR

Our AFLP based NJ consensus phylogeny (Fig 3) places the EAR as sistergroup to the riverine austrotilapiines, an ancestrally monophyletic group of south-central African haplotilapiine clade, which stands apart from almost all East African cichlid clades in its riverine members of the genera *Tilapia* sensu stricto, namely *Chilochromis*, *Congolapia* (Tilapiini) and Steatocranini [2,44]. However, it failed to recover a monophyletic EAR with strong statistical support, because of the comparatively weakly supported placement of the Lake Tanganyika endemic *Trematocara* as the sistergroup to all riverine austrotilapiines and the remaining EAR members. Riverine austrotilapiines formed a weakly supported monophylum (Fig 3, node B1: BS 55) containing *Steatocranus*, *Congolapia*, and *Chilochromis* on the one hand and *Tilapia* sensu stricto on the other hand. The remaining non-*Trematocara* members of ancient Tanganyika tribes (Boulengerochromini (*Boulengerochromis*) and the strongly supported monophylum (BS 100) of Bathybatini and Hemibatini) together formed a weakly supported monophylum (Fig 4, node B: BS 59), which is sister to all remaining EAR members, but also with low node support (Fig 3, node C: BS 44). In summary, the consensus AFLP phylogeny supported a sistergroup relationship of riverine austrotilapiines and most ancient LT tribes with the remaining LT and EAR cichlids, but monophyly of the EAR as well as of basal austrotilapiine subgroups was only weakly supported. Multiple splits in the NeighborNet (Fig 8) not only within riverine austrotilapiines but also between most ancient LT tribes and riverine austrotilapiines further supported ambiguity of phylogenetic signal in the nc multilocus (AFLP) dataset. It is noteworthy in this context, that all previous DNA sequence studies, which had recovered a monophyletic EAR had not included Tilapiini or Steatocranini [5,6,9,82] (Fig 1) nor *Trematocara* [2,49,82]. The only previous molecular support for a monophyletic EAR comes from five SINE-insertions that are present in EAR members, but not in the single studied riverine austrotilapiine species, *Steatocranus casuarius* [83,84]. Using HET removal experiments and nMDS, we assessed the hypothesis that low BS support for a riverine austrotilapiine monophyly (Fig 3, node B1), EAR monophyly (Fig 3, node B) and a sistergroup relationship of most ancient LT tribes with remaining EAR (Fig 3, node C) is rather due to little phylogenetic signal or due to ancient gene flow between selected groups. Indeed, removal experiments revealed a strongly increased monophyly signal for EAR (Figs 3 and 6, node C: BS 44 to 98) as well as for the monophyly of *Boulengerochromis*, *Bathybates and Hemibates* (Figs 3 and 6, node C1: BS 59 to 91) if all riverine austrotilapiines had been removed *in toto*. Strongly increased support was simultaneously detectable across the overall austrotilapiine monophyly (Figs 3 and 6, node A: BS 83 to 92(94)). Removal of single riverine austrotilapiine taxa or taxon groups equally led to strong increase of monophyly signal for nodes B, B1, B2, B3, C and C1 (Figs 3 and 6). Pronounced effects on basal EAR node support was evident especially after removal of the substrate-brooding *Chilochromis duponti*, which is endemic to the Niari-Kouilou, a coastal basin west of the Congo basin: its removal resulted in a strong BS support increase for the monophyly of the three most ancient LT lineages: *Boulengerochromis and Bathybates*, *Hemibates* (Figs 3 and 6, node C1: BS 59 to 91), the monophyly of the EAR without the Bathybatini, Hemibatini and *Boulengerochromis* (Figs 3 and 6, node C: BS 44 to 76(77)), the monophyly of remaining riverine austrotilapiines (Figs 3 and 6, node B1: BS 55 to 86(87)) as well as for the monophyly between just the Congo basin endemics *Steatocranus* and *Congolapia* (Figs 3 and 6, node B2: BS 35 to 85(86)). Analogously, removal of *Tilapia* sensu stricto evoked a strong increase of the monophyly between the Congo basin endemics *Steatocranus* and *Congolapia* (Figs 3 and 6, node B2: BS 35 to 74) but also of all austrotilapiines except *Trematocara* (i.e. riverine austrotilapiines and EAR Figs 3 and 6, node B: BS 57 to 77).

Removals of taxa from the the opposite geographical end, i.e. of the most anciently diverging LT taxa *Boulengerochormis*, Bathybatini, Hemibatini and *Trematocara* as single taxa, or in combination, elicited pronounced effects both for the basal EAR nodes support as well as for the phylogenetic relationships of riverine austrotilapiines with the EAR. Removal of the mouthbrooding *Trematocara* (also in combination with *Bathybates*, *Hemibates* and *Boulengerochromis*) resulted in a very strong increase of the support for the monophyly of riverine austrotilapiines and the EAR (Figs 3 and 6, node B: BS 57 to 94(95)) but also for the monophyly of the EAR without the most ancient LT taxa (Figs 3 and 6, node C: BS 44 to 71(72)). Removal of the substrate-brooding *Boulengerochromis* (alone or in combination with removal of Bathybatini, Hemibatini and *Trematocara*) had a strong impact on node support for the monophyly of riverine austrotilapiines *in toto* (Figs 3 and 6, node B1: 55 to 90(92)), but also an increase of node support for the monophyly of EAR (excl. *Trematocara*), i.e. of *Bathybates* and *Hemibates* with the EAR (Figs 3 and 6, node C: BS 44 to 67(71)). Interestingly, removal of members of the most ancient LT tribes only affected higher level EAR monophyly (Figs 3 and 6, node D (MVhL-clade of Takahashi et al.) [14]: BS 88 to 100), if these were removed *in toto*, hereby supporting the view that *Boulengerochromis*, Bathybatini, Hemibatini and *Trematocara* contain substantial common genetic variation shared only with the MVhL-lineage.

The nMDS projections supported the high similarity of riverine austrotilapiines, Bathybatini and Hemibatini, *Boulengerochromis*, Lamprologini, and part of the ancient LT mouthbrooders on the first axis of nMDS II (Fig 7). Their placement within the NeighbourNet (Fig 8) also reflected a close relationship of these clades, and the reticulations between them suggest a pattern of ancient interspecific gene flow. An hypothetical non-austrotilapiine component may be reflected by the far distant position of *Trematocara* on nMDS I. MtDNA does not provide sufficient resolution for basal austrotilapiine lineages, except for a tendency (Fig 2, BS 59) to support a monophyletic clade of riverine austrotilapiines.

#### 3.1.3. Support for a mosaic genomic composition of Eretmodini

The NJ AFLP phylogeny as well as the NeighborNet (Fig 8) recovered a monophyletic substrate-brooding clade Lamprologini, sister to a monophyletic clade consisting of all remaining EAR members, excluding the most ancient LT clades discussed before (Fig 3, node D: BS 88). This result supports previous allozyme and ncDNA based results [9,15,18], but is strongly contrasted both by our own as well as previous mtDNA or combined mtDNA/ncDNA based results, which place Eretmodini together with Lamprologini at the base of the secondary EAR radiation (AU-test: p<0.01), either sister to Lamprologini (Fig 1, Fig 2; BS 88) [2,5,6,9,16,49], or to Lamprologini and the remaining EAR (Fig 1) [5,6,46]. A close relationship of Lamprologini and Eretmodini is also mirrored by nMDS results, which exclusively group Lamprologini and Eretmodini on most negative values on coordinate 2 of nMDS I (Fig 7). In contrast, the NJ AFLP phylogeny places Eretmodini together with the riverine ‘Malagarasi-*Orthochromis’* as sistergroups to Haplochromini (Fig 3, node F: BS 80) and also the phylogenetic network (Fig 8) reveals reticulations between the neighbouring, distinct clades of Eretmodini and Malagarasi-*Orthochromis*. This topology is at odds with ncDNA sequence data of Clabaut et al.[9] and Schliewen et al. [48], which place two different species of ‘Malagarasi-*Orthochromis’* at very different positions: alternatively either basal to the EAR (Fig 1, *O*. *uvinzae*) [9], or in derived position within Haplochromini (incl. Tropheini) (*O*. *malagaraziensis*) [9,48], but support for the topology comes from the extended nc marker set of Meyer et al. [18]. In concordance with these findings, a sistergroup relationship of Haplochromini and Eretmodini together with Malagarasi-*Orthochromis* was also detected by NeighborNet (Fig 8), indicating an intermediate position of Malagarasi-*Orthochromis* between Eretmodini and Haplochromini. Three different HET removals resulted in a strong increase of BS support for the ‘Malagarasi-*Orthochromis’*-Haplochomini sistergroup relationship (Figs 3 and 6, node G: BS 53 to 93 after removal of Eretmodini; BS 53 to 72 after removal of *Pseudocrenilabrus* cichlids, also in combination with ‘LML-*Orthochromis*’ and ‘northern-Zambian-*Orthochromis’* BS 53 to 75(76)). The Eretmodini removal experiments, which result in increased support for the ‘Malagarasi-*Orthochromis’*-Haplochomini sistergroup relationship (Fig 6), suggests that Eretmodini share genetic components with either ‘Malagarasi-*Orthochromis’* or Haplochromini of the EAR or both, because only support for clades containing parental taxa is significantly compromised by the mosaic genomic composition of putative hybrid specimens. The results of nMDS IV and V further support this alternative signal by separating Eretmodini together with ‘Malagarasi-*Orthochromis’* from all other AFLP-genotyped specimens (Fig 7) after removal of Lamprologini or ancient LT mouthbrooders respectively. Further support comes from reticulations between Malagarasi-*Orthochromis* and Eretmodini in the NeighbourNet (Fig 8) and from the second best supported alternative AFLP topology, which indicates monophyly of Eretmodini and ‘Malagarasi-*Orthochromis’* (Fig 3, BS 41).

Therefore, both new and previously published data support a mosaic genomic composition of Eretmodini and Malagarasi-*Orthochromis*, and they do not contradict a role for this taxon-pair as “transporters of genomic variation” [34] between ancient riverine cichlid assemblages and inshore LT endemic lineages; this is because both share genomic variation not only with each other, but with different riverine and lacustrine groups (‘Malagarasi-*Orthochromis’* and possibly other ‘*Orthochromis*’ and *Pseudocrenilabrus* cichlids, see section 3.1.6. effects on node G). On both physical and ethological criteria, Eretmodini and Malagarasi-*Orthochromis* appear to have been capable of interbreeding with each other as well as with similar taxa across the lake-river ecotones in the greater LT region. The ecological and behavioural characteristics of these two clades can be invoked to propose an admittedly speculative ancient hybridisation scenario; because both taxa are eco-morphologically highly similar; they are benthic-rheophilic cichlids confined to habitats with strong current, whether caused by lacustrine waves or in the high energy, turbulent zones of stream profiles. Moreover, both these taxa exhibit similar reproduction traits: they are mouthbrooders, sometimes biparental, and always lack egg spots. Finally, both are known to occur in direct juxtaposition with each other and with members of additional lineages (e.g. with riverine Haplochromini or with all shore-dwelling LT cichlid tribes) since their geographical ranges overlap: the predominantly lacustrine Eretmodini occur far downstream along the LT-outflow River Lukuga River out of LT [85], while the riverine habitats of the ‘Malagarasi-*Orthochromis’* are concentrated within inflowing tributaries of the LT basin [86].

#### 3.1.4. Are multiple Haplochromini-lineages ancient hybrid composites?

In the consensus NJ-AFLP phylogeny (Fig 3) the endemic LT Tropheini were recovered as one of four major Haplochromini clades, i.e. (1) the *Pseudocrenilabrus* cichlids incl. ‘New Kalungwishi cichlid’, ‘LML-*Orthochromis’* and ‘northern-Zambian-*Orthochromis’* (2) the serranchromines sensu lato of the Congo basin and southern Africa, incl. ‘*Orthochromis*’ *torrenticola*, (3) the predominantly East African ‘modern haplochromines’ including *Astatoreochromis* and a monophyletic Lake Malawi species flock, and (4) the Tropheini. Each clade was supported with BS>99 (Fig 3) and formed a distinct cluster in the NeighborNet (Fig 8), but their interrelationships were only weakly supported. Cyto-nuclear discordance (Fig 2, all discordant signals: AU-Test: p<0.001), HET (Fig 6) and reticulations in the phylogenetic network (Fig 8) and nMDS (Fig 7) indicated that this low support is at least partially due to ancient gene flow among parapatric or geographically overlapping lineages. MtDNA data supported a sistergroup-relationship of ‘LML-*Orthochromis’* with serranaochromines (Fig 2, BS 87) whereas the ‘LML-*Orthochromis’* and ‘northern-Zambian-*Orthochromis’* together with the *Pseudocrenilabrus* cichlids were monophyletic based on nc data (Fig 3, BS 99). The intermediate position of the ‘LML-*Orthochromis’* and ‘northern-Zambian-*Orthochromis’* between the *Pseudocrenilabrus* cichlids and serranochromines was also evident in the NeighborNet (Fig 8), in all nMDS plots (Fig 7) and in the alternative signal of nc data supporting a monophyly of serranochromines, *Pseudocrenilabrus* cichlids, ‘LML-*Orthochromis’* and ‘northern-Zambian-*Orthochromis’* (Fig 3, BS 30). Further support comes from HET, where an increased support for the monophyly of the *Pseudocrenilabrus* group resulted from removal of ‘northern-Zambian-*Orthochromis’* (Figs 3 and 6, node H2: BS 76 to 97), whilst increased support for the monophyly of the ‘LML-*Orthochromis’* and ‘northern-Zambian-*Orthochromis’* was evoked by the removal of the ‘New Kalungwishi cichlid’ (Figs 3 and 6, node H3: BS 80 to 98). Removal of the *Pseudocrenilabrus* cichlids *in toto* as well as removal of the *Pseudocrenilabrus* cichlids, ‘LML-*Orthochromis’* and ‘northern-Zambian-*Orthochromis’* resulted in increased support for the ‘Malagarasi-*Orthochromis’*-Haplochomini sistergroup relationship (Figs 3 and 6, node G: BS 53 to 72 or 76) respectively) also indicating a mosaic genomic composition of the removed taxa. Furthermore, our mitochondrial phylogeny (Fig 2) as well as previous data strongly supported a sistergroup relationship of Tropheini and “modern haplochromines” exclusive of *Astatoreochromis*, which instead is placed as sister group to the the Tropheini-‘modern haplochromines’ clade. Indeed, HET removal of *Astatoreochromis* alone increased monophyly support for the remaining “modern haplochromines” (Figs 3 and 6, node L: BS 90 to 100), but did not affect node support for the Tropheini-“modern haplochromine” relationship (Fig 3, node J: BS 88). However, the removal of all modern haplochromines plus *Astatoreochromis* increased substantially the node support for Tropheini—serranochromines—remaining Haplochromini (Figs 3 and 6, node I: BS 65 to 83). Together with results of nMDS VI (Fig 7) and the phylogenetic network (Fig 8), which clearly place Tropheini as intermediate between serranochromines sensu lato and modern haplochromines (plus *Astatoreochromis*) these results strongly suggest an ancient hybrid swarm origin of Tropheini, i. e. of the single lacustrine Haplochromini species flock endemic to LT. The biogeographical context underscores support for such an origin, because Lake Tanganyika is geographically intermediate between the Congo basin (serranochromines) and East Africa (Haplochromini with ocellated egg spots), and persistent southern and western affluents have linked LT with the southeastern Congo basin, whilst the Malagarasi River and other eastern affluents would have linked the LT cichlids with the East African modern haplochromine fauna. The high degree of persistence of this connection resides in the proto-Malagarasi River, which linked eastern and western drainage systems before formation of LT [87]. As an interesting side-result, the signal for a sistergroup relationship of north African/middle east *Haplochromis* and a monophyletic Malawi flock was compromised by LT drainage riverine ‘*H*.*’ paludinosus* and *‘H*.*’ burtoni*, i.e. BS support increased after their removal (Figs 3 and 6, node M: BS 77 to 100). This finding complements results of Joyce et al. [33], by showing that additional potential east African precursor lineages of the Lake Malawi haplochromine flock may have experienced introgession.

#### 3.1.5. The “ancient mouthbrooder” radiation in Lake Tanganyika

For the first time, the NJ AFLP phylogeny (Fig 3) identified a comparatively weakly supported monophylum (Fig 3, node E1: BS 38) composed of all LT endemic mouthbrooding lineages, which are not members of Haplochromini (Tropheini), Eretmodini or Bathybatini/Hemibatini, i.e. members of the tribes Cyphotilapiini (incl. *‘Ctenochromis’ benthicola*), Perissodini, Cyprichromini, Limnochromini, Benthochromini and Ectodini. Support for monophyly of this group was not evident in the mtDNA-phylogeny presented here (Fig 2) nor in earlier mtDNA phylogenies (Fig 1) [5,6,9] but also the phylogenetic network approach revealed a distinct cluster of “ancient LT mouthbrooders”. HETs suggest that the monophyly signal for this group has been compromised by a potential hybrid origin of certain members of the ancient LT mouthbrooders, i.e. BS support is strongly increased, if either Ectodini (Figs 3 and 6, node E1: 38 to 95) or Cyphotilapiini (incl. *‘Ctenochromis’ benthicola*) (Figs 3 and 6, node E1: 38 to 66(68)) were removed. This indicates that those two subgroups contain genomic components not exclusively shared with ancient LT mouthbrooders, a result which was also indicated by nMDS I to IV, where Ectodini attained an isolated position with regard to the remaining ancient LT mouthbrooders (Fig 7) and by the NeighborNet placing Ectodini at a marginal position of the “ancient LT mouthbrooder” clade (Fig 8) adjacent to the most ancient LT tribes *Boulengerochromis*, Hemibatini and Bathybatini. And interestingly, within the ancient LT mouthbrooders, *Cyphotilapia* and ‘*Ctenochromis’ benthicola* cluster most proximate to Eretmodini on the first coordinate of nMDS 4(Fig 7). Interrelationships among ancient LT mouthbrooder clades were neither supported in the AFLP consensus phylogeny, too, nor by mtDNA, but HET removal of Cyphotilapiini (incl. *‘Ctenochromis’ benthicola*) elicited a strong increase in support for the monophyly of Cyprichromini, Benthochromini and Perissodini (Figs 3 and 6, node E2: BS 40 to 95(96)); this is also the case after removal of Limnochromini alone or together with Ectodini (Figs 3 and 6, node E2: BS 40 to 90(92) or 92(95) respectively). Further, a strong increase in BS support was evident for the monophyly of Cyphotilapiini (incl. *‘Ctenochromis’ benthicola*) and Perissodini (Figs 3 and 6, node E3: BS 37 to 55) after removal of *Benthochromis*. In summary, there is a strong ancient monophyly signal for a clade of ancient LT mouthbrooders. This signal has most likely become compromised by the inclusion of Ectodini and Cyphotilapiini in this group which might possibly contain genetic components of species not included in our sampling (Ectodini), or with inshore LT mouthbrooders (Eretmodini). These results are partially consistent with previous studies, which either did not provide resolution or strong support for monophyly of the ancient LT mouthbrooders [15,84], or which in some analyses already suggested a phylogenetic relationship of Eretmodini with selected members of the ancient LT mouthbrooders [9,18].

### 3.2. ‘Melting Pot Tanganyika’?

Our novel and reticulate phylogenetic hypothesis for the interrelationships of all endemic LT cichlid lineages, together with a diversity of riverine austrotilapiine lineages, support general concerns about simplified scenarios for the evolutionary origin and diversification of LT cichlids [32]. The hypothesis presented here is informed by consilient evidence for reticulate phylogenetic signals, which point to multiple ancient hybridisation events among primarily divergent lineages. Recurrent introgression appears to have affected the diversification of the LT cichlid assemblage at different stages of its formation. Our combined nc- and mtDNA based results do indeed support the previously published suggestion that the extant LT cichlid assemblage comprises four suites of distinct lineages, which evolved in a corresponding sequence of events: (i) early diverging lineages (i.e. those leading to the founding “most ancient Tanganyika tribes” Boulengerochromini, Bathybatini Hemibatini, Trematocarini), (ii) lineages that diverged significantly later than the “most ancient Lake Tanganyika tribes” (i.e. those leading to Lamprologini and Eretmodini), (iii) the lineages leading to the”ancient LT mouthbrooders” (Perissodini, Cyprichromini, Benthochromini, Cyphotilapiini Ectodini and Limnochromini), and (iv) the comparatively young Tropheini, a sublineage of Haplochromini [3,11,19,31]. The inclusion of more or less allopatrically distributed non-LT cichlid lineages from different parts from south-central and eastern Africa, revealed a pattern of complex and partially reticulate phylogenetic relationships of riverine lineages with extant LT endemic lineages on the one hand, and among endemic LT lineages on the other (summarized in Fig 3).

Because rift lakes are ephemeral landforms, it is a matter of simple logic that endemic lacustrine lineages must ultimately trace back their evolutionary origins to riverine lineages. In this case, there is strong evidence that the well differentiated ancient LT cichlid lineages experienced multiple episodes of gene-flow, introgression and possibly hybrid speciation with ancestors of extant riverine cichlid lineages, as well as among LT lineages (see Fig 1: Genner et al.) [3]. These results demand an alternative and more complex model to explain the origin and spatio-temporal evolution of the extant LT cichlid fauna. Such a model is challenged to evaluate available evidence for: (i) the age of formation (and locations) of genuine lacustrine habitats in the LT region; (ii) for the age and tectonic processes that shaped the drainage network and lake connectivity over time; and (iii) absolute ages of the four principal evolutionary events that established the LT cichlid lineages through the interplay between divergence, radiation and reticulations.

Unfortunately, efforts to identify robust spatio-temporal correlations between episodes of increased mtDNA-cladogenesis within the East African radiation (EAR)—and especially LT cichlids—to major tectonic and climatic events have remained vague. This is mainly because of compounding uncertainties in the geological age of extant LT basin, and even less reliable molecular clock age estimates of endemic lineages. Geologically, the extant lake has flooded a string of rift basins formed within in the Albertine rift (the western branch of the East African Rift system). Significantly, LT comprises three deep central basins (> 750 m depth) [88], which at low Pleistocene water levels separated into three distinct palaeo-lakes [89], as well as the northern (Bujumbura) and southern (Mpulungu) basins [26]. Although the absolute age of the formation of these basins remains unknown [90], the age of any true rift formation can only set a rough estimate on the onset of persistent lacustrine conditions in the graben of interest. Most recent studies based on direct thermochronology and sedimentology constrain true rifting activity in the northern basins, i.e. the possibility to form deep rift lakes, to 5.5 Ma at the earliest and likely younger; only the pre-rift formation tectonic activity in the Albertine rift is dated earlier to about 4–11 Ma [91–94]. In part, this new evidence partly clarifies decades of controversy over the age of LT [90], by setting a younger absolute age on LT grabens, which means that all evolutionary scenarios postulated to date for the LT cichlid flock have overestimated the LT age, because they did not consider the implications of this refined reconstruction of Neogene history of the Albertine Rift. It is arguably misleading to assume the older maximum age estimates of 9–12 Ma equate to the formation of LT central basins: namely, 7–8 Ma for the shallow northern Bujumbura and 2–4 Ma for the southern Mpulungu basin. Although these older estimates enjoy widespread subscription, they are not based on direct dating. It is important to recognize that they are derived from roughly uniform LT sediment-accumulation rates, estimated from radiocarbon dates (ages restricted < 55 Ka) of Late Quaternary sediments, extrapolated over the Neogene [26,95]. However, these sedimentation rates have most likely fluctuated, and were certainly not uniform, being likely higher during the late Miocene/early Pliocene [95], because, alongside major climatic changes (thus altering erosion rates of catchments), episodes of regional tectonics and volcanism [95–97] would have disrupted drainage nets and consequently sedimentation flux over time. Along the western margin of the Kalemie Basin, the modern lake covers a drowned topography comprising submerged river valleys lying > 500 m below the extant lake surface [90,98] which testifies to widespread impacts of Pleistocene rifting across the region, coupled with significant changes in past lake levels.

The accuracy of node ages estimated using a molecular clock to constrain the origin of LT lineages is equally debatable. One important reason resides in contentious calibration points for molecular clock analyses derived from [26], and it is furthermore complicated by persistent uncertainties over the global age of the family Cichlidae. Two opposing hypotheses favor either an early Cretaceous origin of Cichlidae in Gondwanaland (vicariance hypotheses) [3,99–102] or a much later Cenozoic origin, implying a subsequent oceanic dispersal (dispersal hypothesis) [103–105]. Unfortunately, where they constrain molecular clock calibrations, these drastically different estimates of cichlid root age translate into disparate estimates for the origin of the LT cichlid clades. Depending on whether Gondwana fragmentation and/or formation of LT itself are chosen as calibration points, estimated timescales for colonization and radiation of LT differ substantially [2,3,19,22,76,103,106,107]. Several recent studies have tried to overcome this problem by either using time constraints derived from cichlid and non-cichlid teleost fossils [2,3,103], but results are no less disparate. They either point to an origin of the ancient LT precursor lineages at around 20–26 Ma (CI´s spanning 14–66 Ma) when including outgroup ages of very old teleost fossils (> 100 Ma from Azuma et al. [99] in Schwarzer et al. [2]), or to a much younger origin at around 16 Ma (CI 11–22 Ma) when using cichlid fossils [3], or only at around 8Ma (CI: 6.9–9.5 Ma) when using comparatively young non-cichlid percomorph fossils for calibration (<100 Ma) [103]. Consequently, even for the youngest age-estimate, the origin of ancient precursor LT cichlid lineages (our “most ancient LT tribes”: *Boulengerochromis*, Bathybatini, Hemibatini, Trematocarini) predates any true LT-graben formation estimate (maximum 5.5 Ma, see above). Notwithstanding this temporal uncertainty, it can be argued that ancestral cladogenic events occurred either in rivers or shallow palaeo-lakes.

Therefore, before any significant tectonic activity occurred in the East African Rift system, LT precursor lineages older than approx. 5.5 Ma must have lived in rivers and swamps across the landscape later reshaped into the Albertine rift. With the onset of tectonic activity, early rift units located along a major pre-rift lineament were established in the Albertine rift region [95,108,109]. This early rifting stage, the so called ‘Nyanja event’ [92], presumably led to the disruption of a former east-west drainage system, i.e. the proto-Malagarasi river, which is represented today by its extant vestiges—the Lukuga and Malagarasi rivers [87,110,111], and this event separated the eastern Congo basin from the Tanzanian plateau [87]. This disruption must have restricted exchange of riverine faunas between central and eastern Africa. Moreover, because the Tanganyika rift zone straddles the interface between these two areas, it is therefore likely that this isolation also impacted on cladogenesis of proto-LT cichlid lineages. After this phase, continued uplift formed several small, geographically distinct, shallow sediment-depocenters (lakes) of different ages, coinciding with landscape warping of the proto-LT-region [35]. Overall, this evidence suggests the regional landscape comprised a mosaic of multiple shallow, ephemeral lakes and inland deltas, whilst repeated catastrophic tectonism likely also reshaped the regional drainage repeatedly. Significantly, the partial palaeo-environmental reconstruction by Cohen et al. [111] identified two isolated fault-contained rift lakes that persisted within the encompassing swampy basin through a more arid period. The combined geological and palaeo-environmental evidence thus points to all three main Tanganyika basins comprising a wetland mosaic rather than a single lake basin that existed from the Late Miocene until the final formation of the contiguous modern Lake Tanganyika in the Late Pleistocene.

In summary, the combined lines of evidence highlight the significance of reticulate evolutionary processes, together with the major uncertainties in the chronology of both geomorphological and cichlid evolutionary phenomena. In combination they argue against a simple model invoking a strictly intra-lacustrine origin and dichotomous cladogenesis of the endemic LT cichlid fauna. Such a diversification model fails to embrace the biotic and geological complexities of the region’s evolution. Instead, the complexities of both cichlid origins and geomorphological evolution raise interesting challenges. Most importantly, any model invoked to explain the evolution of the extant LT cichlid assemblage must take into account the protracted history of palaeo-drainage dynamics focused around the Tanganyika region. Its complex sequence of events arguably created windows of opportunity for allopatric and allochronic cichlid speciation in rivers and lakes of the proto-LT region on the one hand, and for the emergence of several shallow lake proto-LT-species flock lineages on the other. It must further take into account the evidence, that:

1. repeated tectonic disruptions within the Tanganyika rift zone have likely been important drivers of speciation and hybridisation by breaking and forming links between palaeo-rivers at more local spatial scales, whilst turnovers of lake stages repeatedly provided new ecological opportunities; and more recently alternating with moderately moist interglacials, pulses of Pleistocene aridity increased environmental instability across central Africa. Alongside drainage and basin-rearrangements, repeated climatic changes in lake levels would have opened up multiple opportunities for secondary contact between primarily allopatric palaeo-lake and riverine lineages.
2. the extant LT basin may have served, repeatedly, as a ‘melting pot’ of originally divergent proto-LT cichlid lineages.

## Conclusion

Despite major uncertainties in the estimated timings of cichlid cladogenesis, as well as the tempo and mode of the tectonism that has reshaped landscapes across the LT region, it has been assumed that we can use an explicit, relatively parsimonious model to explain the origins and diversification of the LT cichlids, by linking the origin of these fishes to the first appearance of one substantial, ancient rift lake anchored in turn by straightforward geochronology. However, recent progress in both ichthyology and geology reveals that the evolution of both biota and landscape are much more complex than previously assumed. Not only does this complexity hinder progress to elucidate explicit determinants of biotic evolution, but it also complicates attempts to reconstruct explicit details of rifting, landscape evolution, and associated lake basin formation. Nevertheless, there is convincing evidence that the answers are to be found in the ancestral wetlands of the region, reconstructing how the dynamics of landscape evolution reshaped lakes and rivers to culminate in the geomorphological and biological attributes of the extant LT basin. Together with the genomes of organisms that evolved in the greater LT region, lacustrine sediments and related geological formations are the evolutionary archives to search for the answers. These circumstances present exciting challenges for research seeking to reconstruct the accurate evolutionary history of LT biodiversity and their palaeo-environments. It appears that the details of cichlid evolution are even more complicated, when we acknowledge how Neogene palaeo-drainage dynamics beyond the greater LT region, i.e. across south-central and eastern Africa, caused extensive interspecific gene flow. It is equally important to single out, and (where feasible fix) limitations of available methods, particularly in uncertainties in molecular clock calibrations that currently hamper robust node age estimates of cichlid evolution. The significance of this problem is underscored by the complexities of the LT cichlid radiation as revealed here, and it particularly argues against attempting to invoke the age of a truly lacustrine environment as a molecular dating constraint.

## Supporting Information

S1 Table Complete list of specimens used for AFLP and ND2 approach. (XLS)
