# Supplementary figures and images for: Correction: Lake Tanganyika—A 'Melting Pot' of Ancient and Young Cichlid Lineages (Teleostei: Cichlidae)?
Source: PLoS One. 2015 Jul 15;10(7):e0132615. doi: 10.1371/journal.pone.0132615 (PMC4503626; doi:10.1371/journal.pone.0132615)

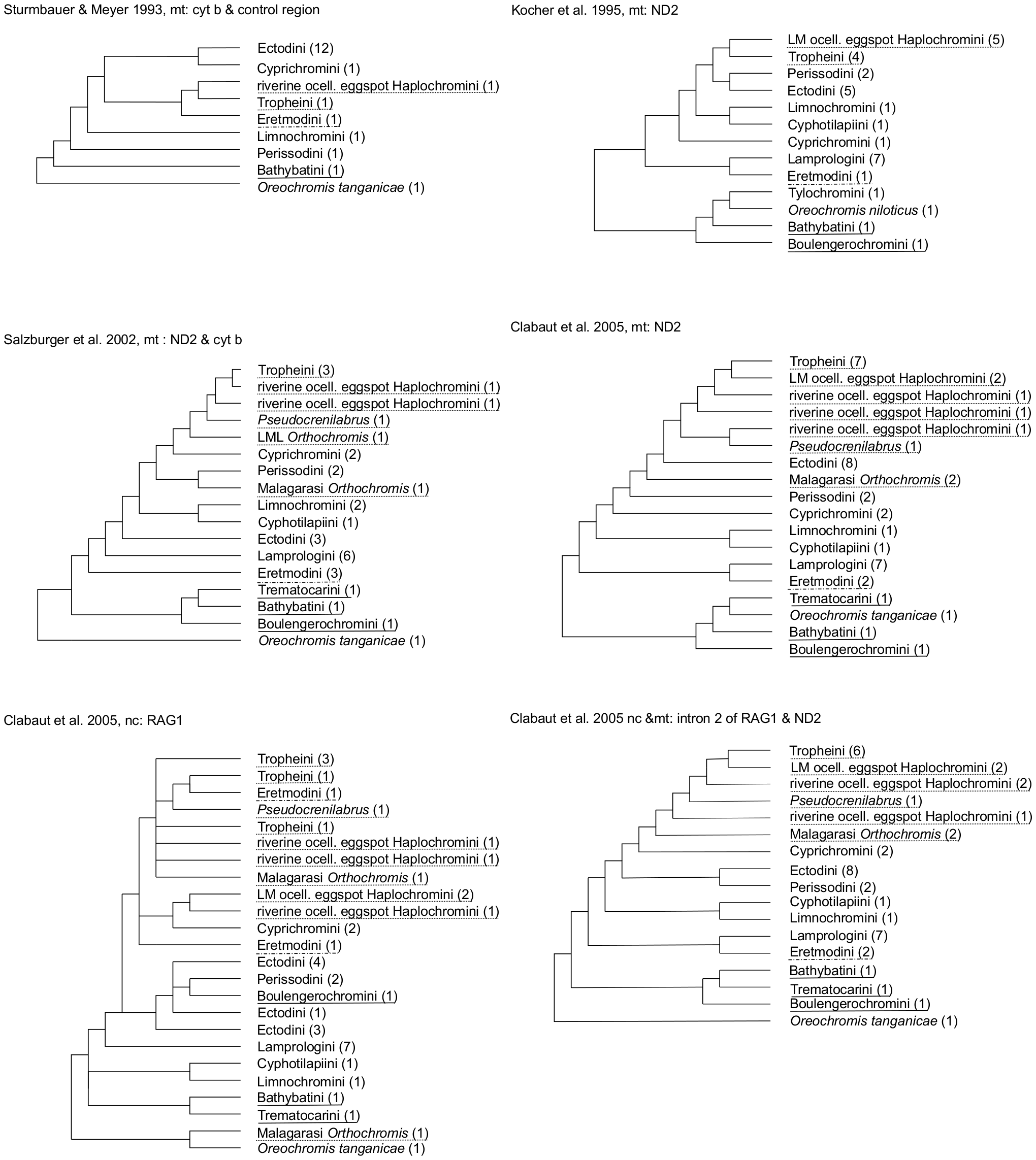

Supplement: S1 Fig — Tribes with ambiguous placement are highlighted as follows: most ancient Tanganyika tribes (continuous line); Eretmodini (dashed line); haplochromine lineages (dotted line). Numbers in brackets correspond to the number of individuals. (TIF) [file pone.0132615.s001.tif]

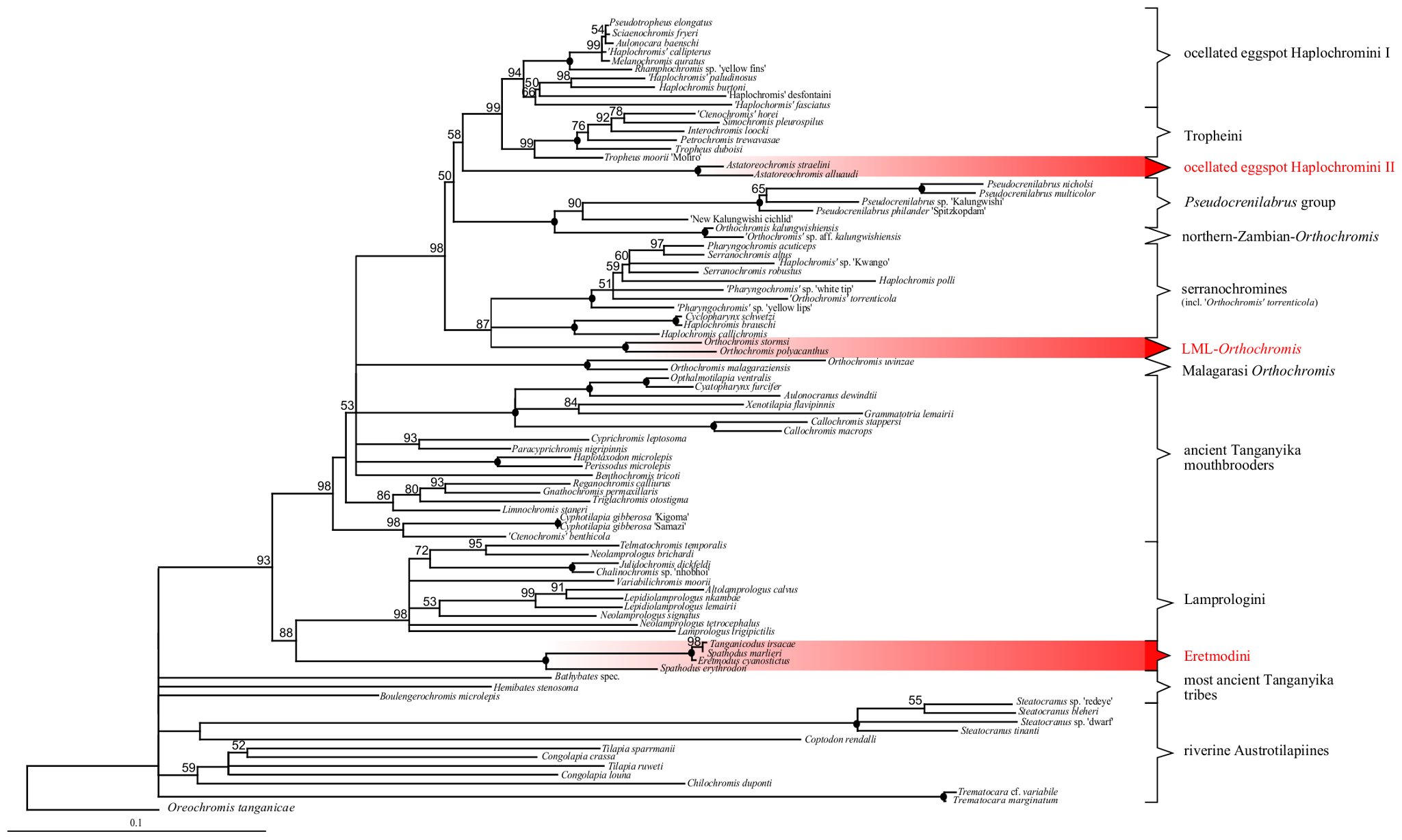

Supplement: S2 Fig — The topology of the best scoring ML tree is based on ND2 sequences (1010bp). Numbers at nodes refer to bootstrap-values (BS 500 replicates), 100% BS support is indicated by filled, black circles. Significant deviations from the nc-based NJ-topology are highlighted in red. (TIF) [file pone.0132615.s002.tif]

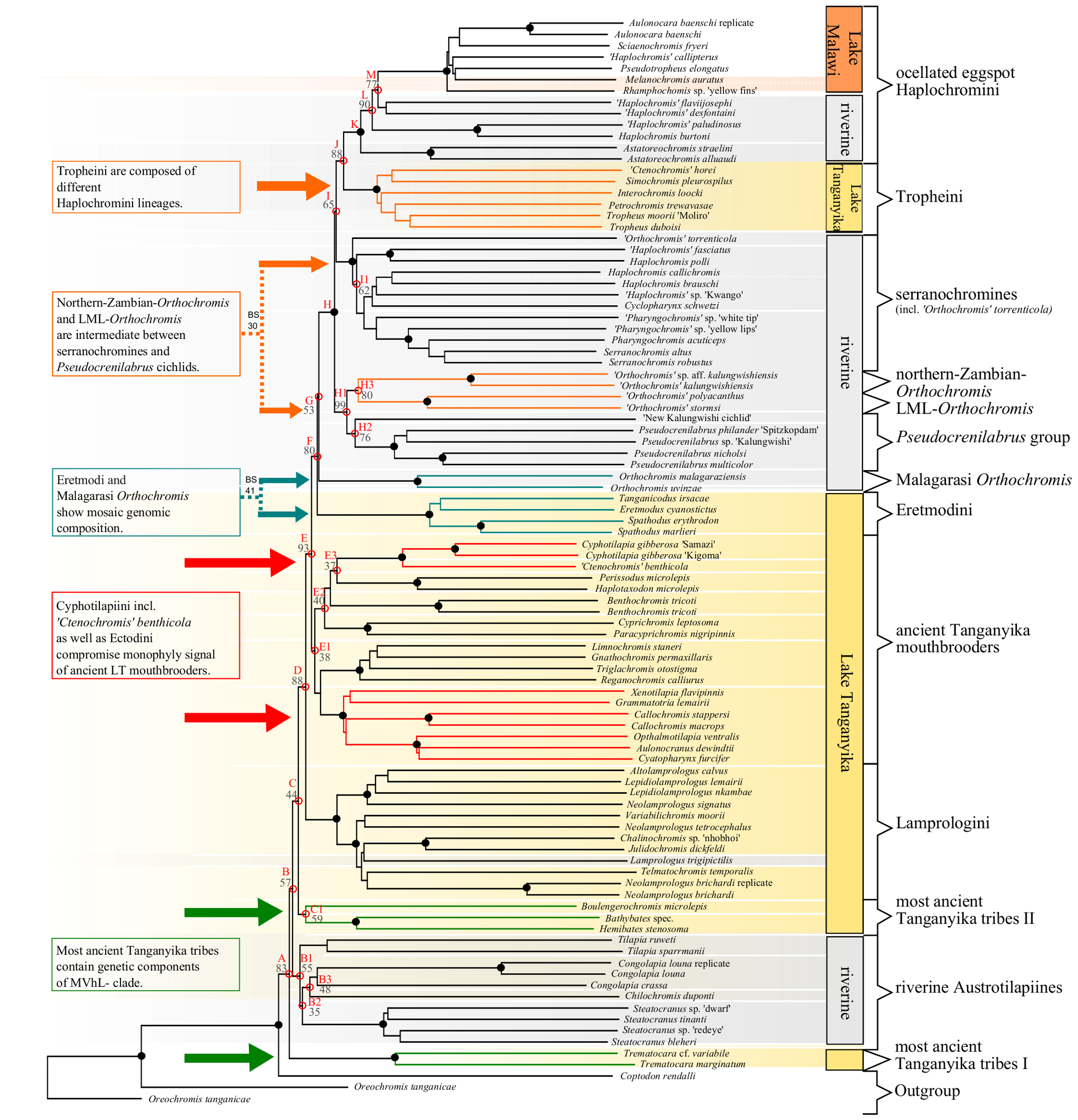

Supplement: S3 Fig — The NJ consensus topology is based on Jaccard’s distances [62] of 3312 nc loci. Nodes affected by homoplasious effects are designated with letters A-M and indicated by open, red circles. Numbers at nodes refer to bootstrap-values (BS 500 replicates) and a 100% BS support is indicated by filled, black circles. Geographic distribution of taxa is depicted vertically on the right and colour shaded in the tree (Lake Tanganyika: yellow; Lake Malawi orange; rivers: grey). Major effects detected with HET and inference of cyto-nuclear discordances are delineated in coloured boxes on the left and correspond to those in Fig 6 and Fig 8. Arrows and coloured branches point to clades which especially introduce homoplasy in the dataset. Strong alternative signal (BS support >30) is denoted with dotted lines on the left. (TIF) [file pone.0132615.s003.tif]

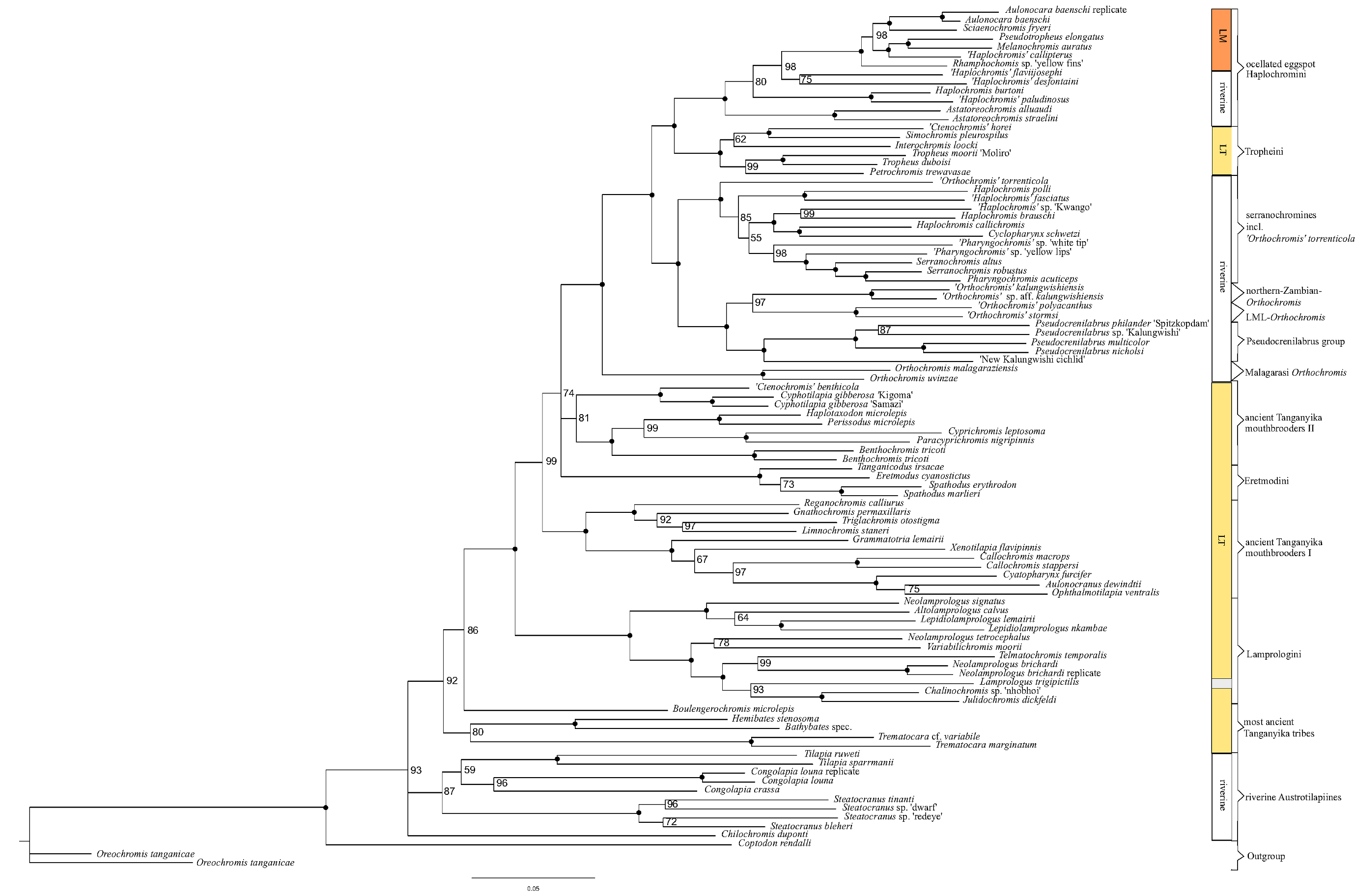

Supplement: S4 Fig — Consensus topology with branch support values depicted at nodes, dots correspond to 100% Bayesian Posterior Probability. (TIF) [file pone.0132615.s004.tif]

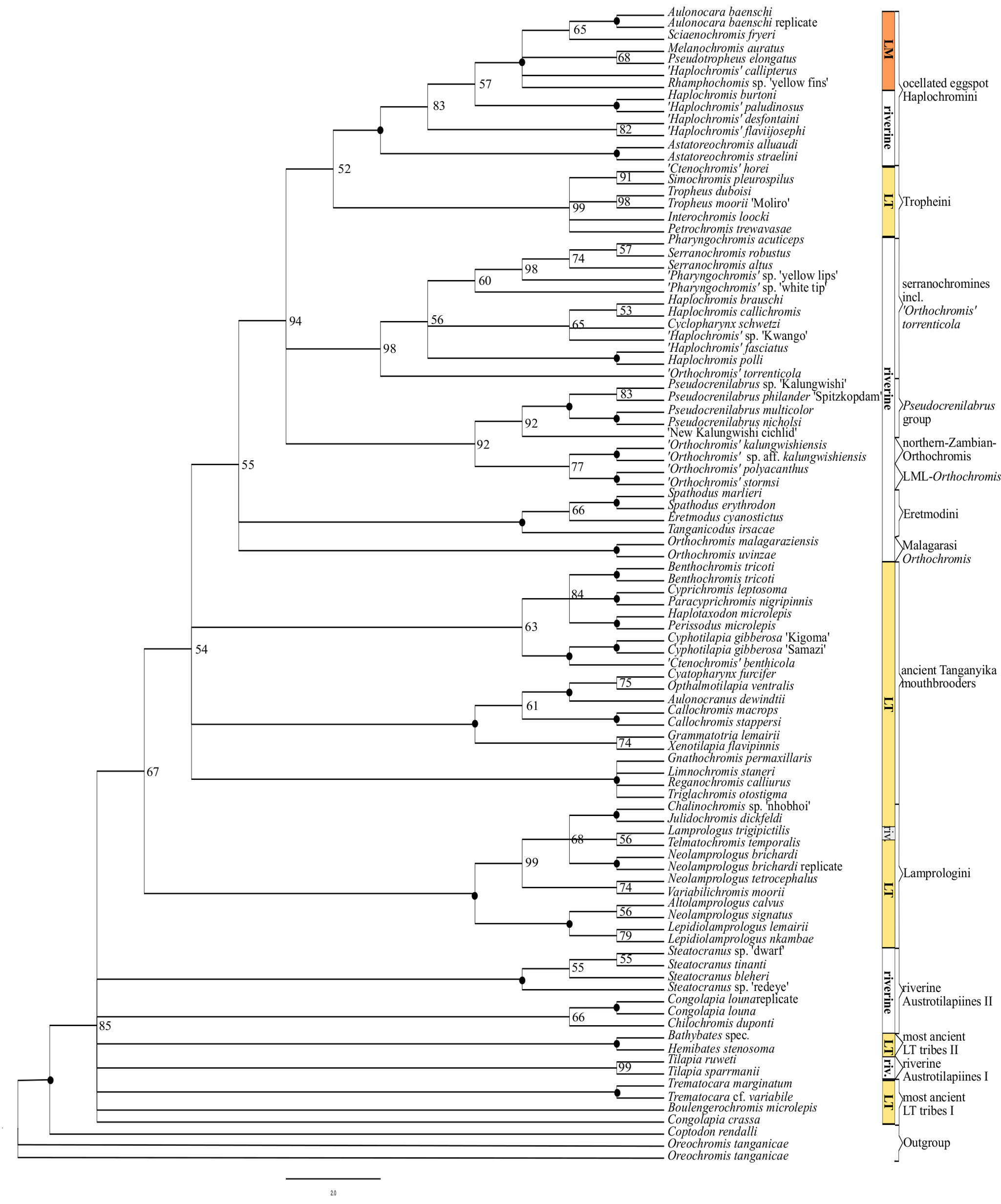

Supplement: S5 Fig — 50% majority rule consensus topology of 1000 BS replicates. BS support is depicted at respective nodes, dots correspond to 100 BS support. (TIF) [file pone.0132615.s005.tif]

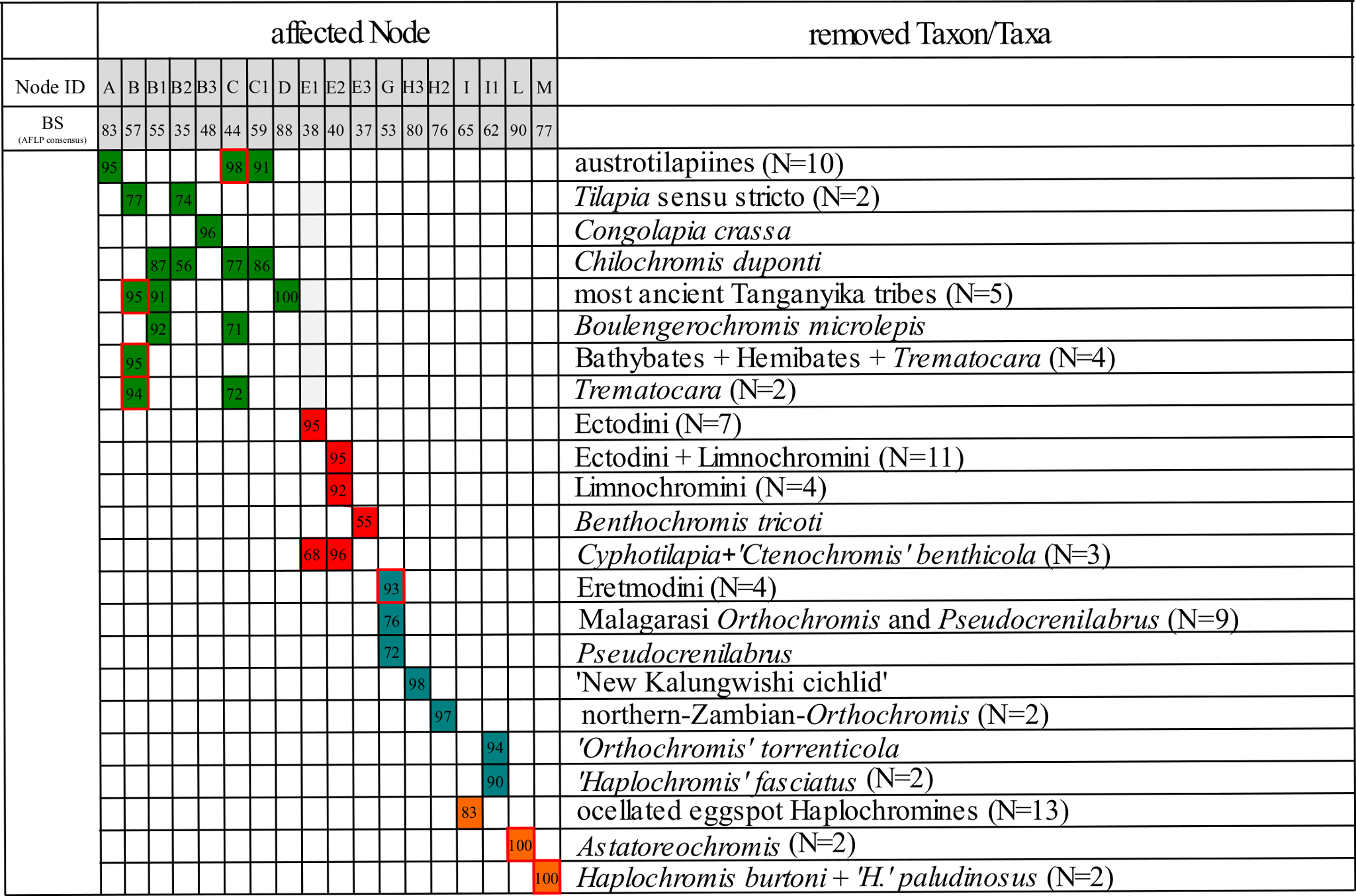

Supplement: S6 Fig — For all affected nodes the bootstrap (BS) support in the consensus AFLP topology as well as BS support after the influential removals is shown and removed taxa are specified. Node IDs correspond to those in the nc NJ-consensus phylogeny (Fig 3). Single effects were grouped according to four major effects and are represented by different colours. Potentially artificial BS increases due to ‘support carryover’ (see methods) are highlighted with red frames. (TIF) [file pone.0132615.s006.tif]

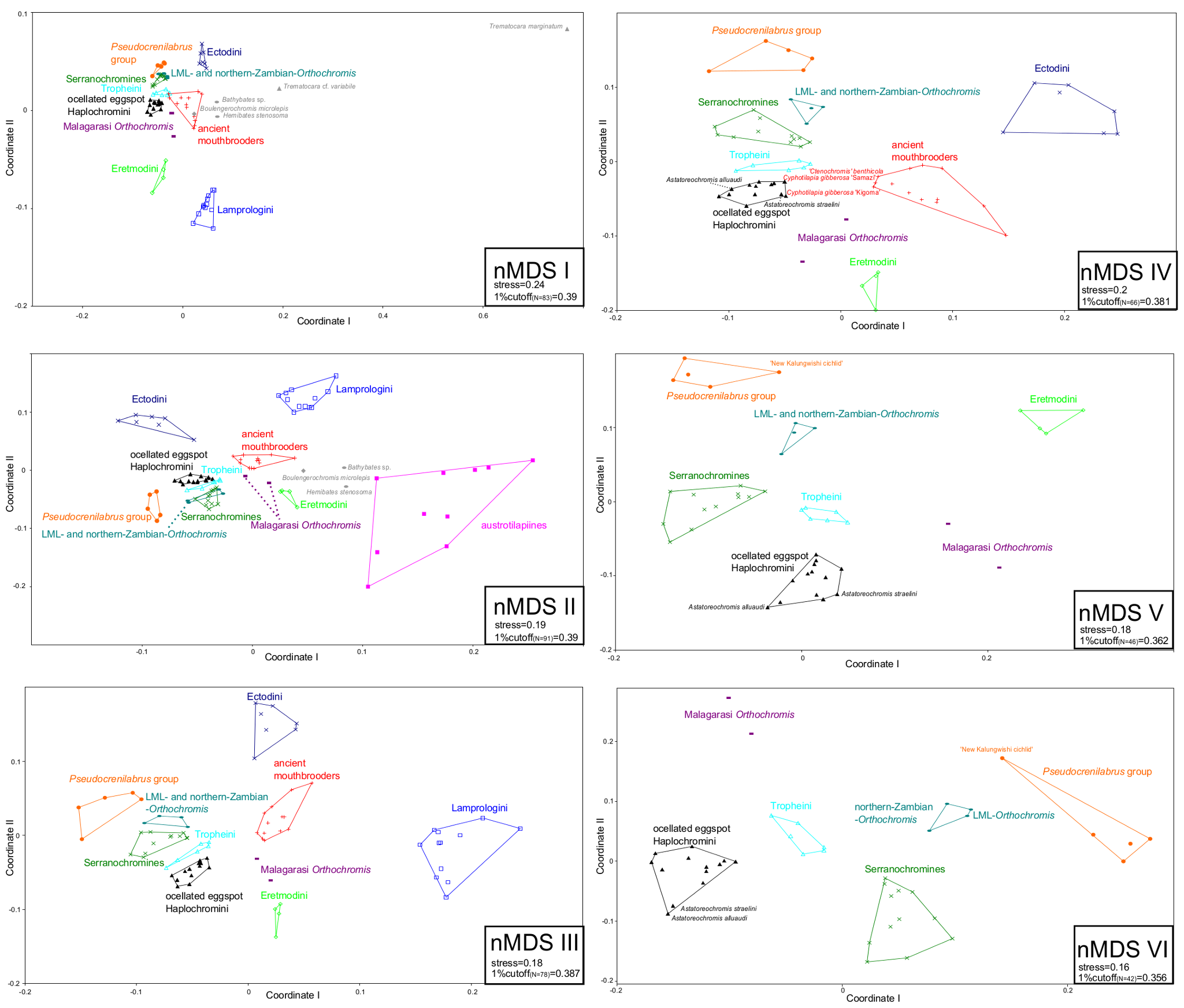

Supplement: S7 Fig — To infer phylogenetic relationships in an altered variance space, major sistergroups of ingroup taxa according to the consensus NJ topology were stepwise eliminated. NMDS plots are based on Jaccard´s distances [62] of nc (AFLP) data. Kruskall’s stress values as well as the corresponding 1% cutoff values [74] are given for each projection. (TIF) [file pone.0132615.s007.tif]

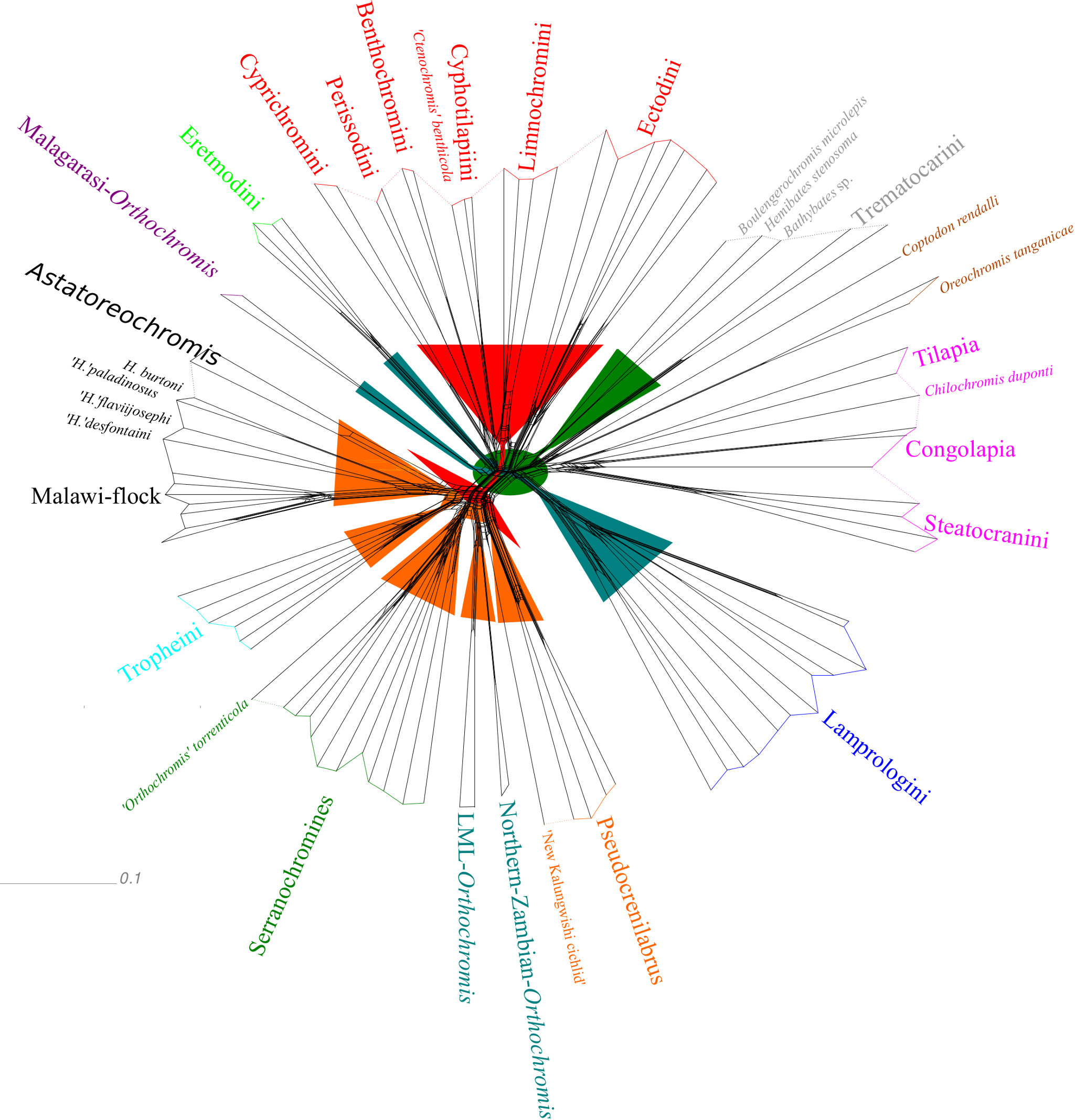

Supplement: S8 Fig — The NeighborNet network topology is based on Jaccard’s distances [62]. Specimens of distinct lineages are grouped and informal groups used in this work are depicted in different colours. Funnels highlight conflicting signal corresponding to the four major effects detected with HET and inference of cyto-nuclear discordances and are coloured according to Fig 3 and Fig 6. (TIF) [file pone.0132615.s008.tif]
